# Intervention targeting and retention, engagement and abstinence outcomes among Latino and White users of Smokefree.gov text messaging programmes: a cross-sectional study

**DOI:** 10.1136/bmjph-2023-000222

**Published:** 2023-11-08

**Authors:** Sherine El-Toukhy, Kristyn Kamke

**Affiliations:** 1 Division of Intramural Research, National Institute on Minority Health and Health Disparities, Bethesda, Maryland, USA

**Keywords:** public health, epidemiology, statistics and numerical data

## Abstract

**Background:**

We examined retention, engagement and abstinence among Latino users of SmokefreeTXT en Español (SFTXTesp), a Latino-targeted text messaging smoking cessation intervention, and Latino and White users of SmokefreeTXT (SFTXT), a non-targeted intervention.

**Methods:**

Data came from 12 281 users (1562 Latino SFTXTesp users and 2301 Latino and 8418 White SFTXT users). We conducted time-to-drop-out analyses by race/ethnicity. Using logistic regression, we examined associations between intervention targeting, race/ethnicity and responses to smoking status prompts, an engagement metric, and self-reported abstinence on quit day, intervention end and 1-month follow-up. Age, gender, census region, smoking frequency, cigarettes smoked per day, prequit time and number of quit attempts were covariates.

**Results:**

Latinos in SFTXTesp (adjusted OR, aOR 0.63) and SFTXT (0.88) were less likely to drop out of the intervention than Whites. SFTXTesp Latino users had higher response rates to smoking prompts than SFTXT Latinos users (aORs 1.35, quit day; 1.84, intervention end; 1.82, 1-month follow-up). However, SFTXTesp and SFTXT Latino users had lower response rates than Whites (aORs 0.68, 0.45, quit day; 0.60, 0.30, intervention end; 0.64, 0.33, 1-month follow-up). Abstinence was equivalent among Latinos in SFTXTesp and SFTXT interventions, but Latinos using SFTXTesp and SFTXT were less likely to be abstinent than Whites (aORs 0.42, 0.41, quit day; 0.45, 0.37, intervention end and 0.53, 0.35, 1-month follow-up).

**Conclusion:**

Linguistic intervention targeting improved retention and engagement among Latinos, but not abstinence. Latinos had higher retention but lower engagement and abstinence rates than Whites. Cultural targeting may engage Latinos in smoking cessation interventions and improve abstinence.

**WHAT IS ALREADY KNOWN ON THIS TOPIC**

**WHAT THIS STUDY ADDS**

- The study is a real-world assessment of Smokefree.gov text messaging smoking cessation interventions. SmokefreeTXT en Español, a Latino-targeted text messaging intervention, improved retention and engagement among Latino users compared with Latino users of SmokefreeTXT, a non-targeted intervention. Intervention targeting did not affect abstinence. Despite their lower retention rates, Whites had better engagement and abstinence outcomes than Latinos who used either the targeted or non-targeted intervention.

**HOW THIS STUDY MIGHT AFFECT RESEARCH, PRACTICE OR POLICY**

- Beyond linguistic targeting, cultural adaptations of text messaging smoking cessation interventions are needed to improve retention, engagement and abstinence outcomes among Latino smokers and reduce smoking and cessation disparities with Whites.

## Introduction

 Latino cigarette smokers are a unique population for tobacco treatment efforts. Constituting 8.8% of US adult smokers,^1^ they are a heterogeneous group that shows considerable within-group variations in smoking prevalence, frequency, intensity and quit rates attributable to several factors (eg, nationality).^2^ Tobacco-related illnesses are leading causes of death among Latinos.^3^ Latinos have lower prevalence of being asked about tobacco use, receiving quit advice from healthcare providers and using smoking cessation pharmaceutical aids or counselling resources.^4 5^

Text messaging smoking cessation interventions are self-help programmes that provide smokers with evidence-based treatment to help them quit or reduce cigarettes smoked.^6^,^12^ Text messaging is a viable intervention platform for Latinos among whom cellphone ownership is 100% (85% for smartphones, 14% for cellphones only).^13^ These interventions offer several benefits, including reduced cost, time and stigma associated with their use.^9^ Few text messaging smoking cessation interventions target Latinos.^14^

Targeted interventions appeal to predefined subgroups with shared characteristics, usually static or slow-changing characteristics, such as race/ethnicity.^15^ Targeting is achieved through various strategies—from linguistic to cultural adaptations.^16^ Targeted interventions are more efficacious in reducing smoking than non-targeted interventions.^16^ Gaps remain in our understanding of the efficacy and effectiveness of Latino-targeting smoking cessation text messaging interventions. For example, Quitxt SMS,^17^ an intervention targeting Latinos aged 18–29 in Southern Texas, Latino Kick Buts,^18^ an adaptation of Txt2Stop intervention,^19^ and Vive sin Tabaco… ¡Decídete!,^20^ an intervention targeting Mexican smokers, have not been evaluated on a wide scale. Furthermore, evaluations of targeted interventions have been limited to examining whether targeting improves outcomes within their target population.^16^ Although this approach can show superiority of targeted (vs non-targeted) interventions among the target population, it does not show whether targeting reduces existent cessation disparities between minority and majority groups.

SmokefreeTXT en Español (SFTXTesp) is a free and publicly available smoking cessation intervention for Latinos through the Smokefree.gov Initiative of the National Cancer Institute (NCI). SFTXTesp is a linguistic adaptation of NCI’s general text messaging smoking cessation intervention, SmokefreeTXT (SFTXT), which is available in English for US smokers.^21^ Latinos can enrol in SFTXTesp or SFTXT, whereas other races/ethnicities can enrol in SFTXT. Both interventions are nearly identical in intervention duration, message content and structure, which offers a unique opportunity to examine the effects of intervention targeting on intervention outcomes among Latinos and Whites.

In this observational study, we examine associations between (a) targeting and intervention outcomes among Latinos in SFTXTesp and SFTXT and (b) race/ethnicity and intervention outcomes among Latinos in either SFTXTesp or SFTXT and Whites in SFTXT. The primary outcomes were retention by intervention end, engagement and point prevalence abstinence on quit day (ie, day 0), intervention end (ie, day 42), and 1-month postintervention end (ie, day 72). Retention is when a participant completes an intervention,^22^ whereas engagement is an investment of physical, affective and cognitive energy in a stimulus/task.^23^ Retention and engagement are important determinants for intervention efficacy and achieving abstinence.^2224^,^27^

## Methods

### Interventions description

SFTXTesp and SFTXT, available only to smokers in the USA, are 6–8 weeks in duration and follow an identical structure. At signup, users select a quit day, which is recommended to be within 2 weeks of signup. During this optional preparation phase, users receive messages to prepare them to quit smoking.^28^ Starting on quit day, users receive messages that include cessation tips and motivational content over 42 days.^28^ Users receive 3–5 daily messages on average, with more messages delivered in the treatment phase (ie, weeks 1–2) than in the maintenance phase (ie, weeks 3–6). Users are unable to alter the number of messages they receive. At any time after signup, users can reset their quit day, opt-out of the intervention and text keywords to receive additional on-demand content. Users receive smoking status prompts, weekly throughout the intervention starting on quit day (ie, day 0) until intervention end (ie, day 42), and at 1, 3 and 6-month postintervention completion (ie, days 72, 132, 222).

### Sample

Data came from eligible records of Latino SFTXTesp users and of Latino and White web enrollees in SFTXT who had complete race/ethnicity information, spanning from 1 January 2016 to 31 December 2018. Records represented real-world SFTXTesp and SFTXT users who self-selected to enrol in either intervention. We deleted duplicate, inconsistent and incomplete records (online supplemental table 1). Specifically, we used the most recent quit attempt for users who reset their quit days. We excluded users whose quit day preceded their signup date, who opted out of the intervention before the prequit period, and who set a quit day less than 42 days prior to end-of-study date (ie, 31 December 2018 when data were pulled). We applied these exclusion criteria to each dataset, resulting in an analytical sample of 12 281 unique records.

### Measures

Users reported their age, gender, smoking frequency (daily vs non-daily), cigarettes smoked per day and zip code. SFTXT users had a ‘non-binary’ or ‘prefer not to say’ gender option plus male and female. Daily smokers reported smoking ‘every day’, whereas non-daily smokers reported smoking ‘most days’, ‘some days’, or ‘less than that’. Cigarettes smoked per day were classified into light (≤10 cigarettes), moderate (11–20 cigarettes) and heavy (>20 cigarettes). Residential zip codes were converted into four US census regions plus Puerto Rico and the Virgin Islands. SFTXT users who enrolled online reported their race/ethnicity. SFTXT users who self-identified as Latinos were considered as such regardless of race. All SFTXTesp users were considered Latinos.

Users’ signup date, quit day and, if applicable, opt-out date and quit day reset(s) were automatically captured. We used these dates to calculate prequit time and time-to-drop-out. For users whose signup to quit day was ≤14 days, prequit time equaled their time from signup to quit day. For users whose quit day was >14 days, we reset their prequit time to 14 days because users receive messages 14 days prior to quit day. Prequit time served as a covariate to adjust for potential effects of the preparation phase and intervention dose.^9 29^ Time-to-drop-out ranged from 0 to 42 and was used in the survival analysis among intervention initiators (ie, users who made it to quit day).

We created three dichotomous variables: intervention completion status, smoking status and response status. For completion status, users who opted out by texting ‘STOP’ or for whom text messages were undeliverable between day −14 (ie, 14 days before quit day) and intervention end (ie, day 42) were considered ‘non-completers’, whereas users who remained in the intervention until day 42, regardless of intervention engagement, were ‘completers’. For smoking status, users were considered ‘smokers’ if they responded ‘yes’, and ‘non-smokers’ if they responded ‘no’ to each smoking status question (ie, ‘Day 1 is almost over. Have you smoked today?’ on quit day, ‘Hi there! Have you smoked in the past 7 days?’ thereafter, and ‘Hi, just checking in. Have you smoked in the last 30 days?’ on 1-month follow-up). They were considered ‘responders’ if they answered the smoking status question with either a ‘yes’ or a ‘no’ and ‘non-responders’ if they did not respond. Response to smoking status questions captured user engagement and abstinence.

### Data analysis

SFTXTesp had 38.67% (n=604) missing data on five sociodemographic variables, whereas SFTXT had 0.57% (n=61) missing data on three variables. Missingness was not completely at random (SFTXTesp χ^2^=39.45, p=0.009; SFTXT χ^2^=54.18, p<0.001). To avoid introducing bias with listwise deletion, we performed 20 imputations where variables with no missing values in each dataset were used as covariates. Users whose self-reported age fell outside 13–99 years (n*=*52) were considered missing and imputed. Response and abstinence statuses were not imputed.

To examine retention, we conducted a Cox regression of time from quit day to drop-out. For engagement and abstinence outcomes, we ran logistic regression models among Latino users in SFTXTesp or SFTXT (SFTXT Latino users were reference group) and among Latinos and Whites in SFTXTesp or SFTXT (SFTXT White users were reference group) at all assessment time points (ie, days 0, 7, 14, 21, 28, 35, 42 and 72). In each engagement model, n was the number of users who remained in the intervention on the day smoking status was assessed. In the abstinence models, users who failed to respond to smoking status prompts and/or had dropped out were considered smokers following an intention-to-treat approach. All analyses were limited to intervention initiators and were adjusted for age, gender, census region, smoking frequency, cigarettes smoked per day, prequit time and number of quit attempts. Day 72 engagement and abstinence models were further limited to those who could have made it to day 72 by end-of-study date and, thus, could have received the smoking status prompt. As sensitivity analyses, we ran all analyses among users who completed the intervention (ie, intervention completers) and among users without missing data (ie, complete case) where intervention outcomes are stronger.^8^

### Patient and public involvement

Participants were not involved in the design, conduct, reporting or dissemination of this study.

## Results

Sample characteristics for full dataset and complete case appear in online supplemental tables 2 and 3.

### Retention

**Figure 1:**
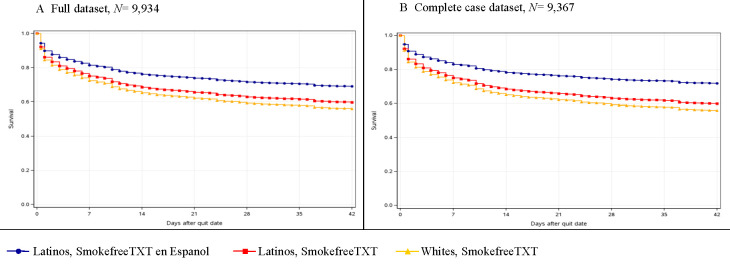
Survival analysis of time-to-dropout among SmokefreeTXT en Español (SFTXTesp) and SmokefreeTXT (SFTXT) Latino and White intervention initiators. Analyses were adjusted for age, gender, region, smoking frequency, cigarettes smoked per day, prequit time and number of quit attempts.

Drop-out before quit day ranged from 28.69% to 52.72% among SFTXTesp and SFTXT Latino users (online supplemental table 2). Intervention non-initiators (ie, those who dropped out before quit day) were more likely to be males and SFTXT Latino users, smoke heavily and have 2+ quit attempts (online supplemental table 4). Time from quit day to drop-out averaged 10.40, 6.64 and 8.73 days among SFTXTesp Latino users, SFTXT Latino users and SFTXT White users (online supplemental table 2). Latinos in both SFTXTesp (adjusted OR, aOR 0.63) and SFTXT (aOR 0.88) were less likely to drop out compared with Whites in SFTXT (table 1, figure 1A). At intervention end, 68.9% and 59.6% of Latino SFTXTesp and SFTXT users were retained compared with 55.8% SFTXT White users. Similar patterns emerged under complete-case analysis (table 1, figure 1B).

**Table 1:** HRs for drop-out among SmokefreeTXT en Español (SFTXTesp) and SmokefreeTXT (SFTXT) intervention initiators

	Full dataset (N=9934)			Complete-case dataset (N=9367)		
	HR	LCL	UCL	HR	LCL	UCL
Age	**0.99**	**0.99**	**0.99**	**0.99**	**0.99**	**0.99**
Gender (ref: female)						
Male	0.96	0.90	1.02	0.96	0.90	1.03
Other	0.93	0.61	1.42	0.93	0.61	1.43
Region (ref: South)						
Northeast	**0.87**	**0.80**	**0.95**	**0.87**	**0.80**	**0.96**
Midwest	0.93	0.86	1.01	0.94	0.86	1.01
West	0.95	0.87	1.03	0.93	0.85	1.02
Puerto Rico and Virgin Islands	1.06	0.77	1.46	0.98	0.66	1.45
Smoking frequency (ref: non-daily)						
Daily	1.05	0.93	1.19	1.04	0.92	1.18
Cigarettes smoked per day (ref: light)						
Moderate	1.03	0.95	1.12	1.04	0.95	1.13
Heavy	**1.12**	**1.02**	**1.22**	**1.12**	**1.03**	**1.22**
Prequit time	**0.94**	**0.93**	**0.94**	**0.94**	**0.93**	**0.95**
No of quit attempts (ref: 1)						
2+	**1.28**	**1.08**	**1.50**	**1.23**	**1.04**	**1.47**
Race/ethnicity (ref: Whites, SFTXT)						
Latinos, SFTXTesp	**0.63**	**0.57**	**0.70**	**0.56**	**0.49**	**0.65**
Latinos, SFTXT	**0.88**	**0.81**	**0.97**	**0.88**	**0.81**	**0.96**

Bolded cells represent significant results.

LCL, lower confidence level; UCL, upper confidence level

### Engagement

Overall response rate was 21.94% on quit day, 10.94% on day 42 and 14.88% on day 72 (table 2). SFTXTesp Latino users were more likely to respond to smoking status prompts than SFTXT Latino users on quit day, day 42 and day 72 (aORs 1.35, 1.84, 1.82) (table 3). Compared with SFTXT White users, SFTXTesp and SFTXT Latino users were less likely to respond to smoking status prompts across all time points (eg, aORs 0.68, 0.45 on quit day; 0.60, 0.30 on day 42 and 0.64, 0.33 on day 72) (table 4). Intervention completer (online supplemental tables 5–7) and complete case (online supplemental tables 8–10) analyses largely mirrored these results.

**Table 2:** Response rates and point prevalence abstinence among SmokefreeTXT en Español (SFTXTesp) and SmokefreeTXT (SFTXT) intervention initiators (full dataset, N=9934)

	Did not respond, n	Responded		Response rate denominator, n	Response rate, %	Abstinence rate denominator, n	Abstinence, %
		Abstinent, n	Not abstinent, n				
Quit day							
Latinos, SFTXTesp	1122	119	146	1387	19.11	1387	8.58
Latinos, SFTXT	1419	112	73	1604	11.53	1604	6.98
Whites, SFTXT	5213	1205	525	6943	24.92	6943	17.36
Overall	7754	1436	744	9934	21.94	9934	14.46
Day 7							
Latinos, SFTXTesp	955	81	135	1171	18.45	1387	5.84
Latinos, SFTXT	1016	86	68	1170	13.16	1604	5.36
Whites, SFTXT	3601	949	570	5120	29.67	6943	13.67
Overall	5572	1116	773	7461	25.32	9934	11.23
Day 14							
Latinos, SFTXTesp	902	73	113	1088	17.10	1387	5.26
Latinos, SFTXT	969	79	37	1085	10.69	1604	4.93
Whites, SFTXT	3393	827	328	4548	25.40	6943	11.91
Overall	5264	979	478	6721	21.68	9934	9.86
Day 21							
Latinos, SFTXTesp	895	71	78	1044	14.27	1387	5.12
Latinos, SFTXT	958	64	26	1048	8.59	1604	3.99
Whites, SFTXT	3341	746	225	4312	22.52	6943	10.74
Overall	5194	881	329	6404	18.89	9934	8.87
Day 28							
Latinos, SFTXTesp	888	45	65	998	11.02	1387	3.24
Latinos, SFTXT	941	57	25	1023	8.02	1604	3.55
Whites, SFTXT	3307	639	164	4110	19.54	6943	9.20
Overall	5136	741	254	6131	16.23	9934	7.46
Day 35							
Latinos, SFTXTesp	896	37	46	979	8.48	1387	2.67
Latinos, SFTXT	938	50	20	1008	6.94	1604	3.12
Whites, SFTXT	3235	615	129	3979	18.70	6943	8.86
Overall	5069	702	195	5966	15.04	9934	7.07
Day 42							
Latinos, SFTXTesp	870	42	41	953	8.71	1387	3.03
Latinos, SFTXT	947	34	3	984	3.76	1604	2.12
Whites, SFTXT	3328	430	82	3840	13.33	6943	6.19
Overall	5145	506	126	5777	10.94	9934	5.09
Day 72							
Latinos, SFTXTesp	819	52	66	937	12.59	1365	3.81
Latinos, SFTXT	819	33	19	871	5.97	1440	2.29
Whites, SFTXT	2886	444	177	3507	17.71	6431	6.90
Overall	4524	529	262	5315	14.88	9236	5.73

**Table 3:** Correlates of engagement and abstinence outcomes among SmokefreeTXT en Español (SFTXTesp) and SmokefreeTXT (SFTXT) Latino intervention initiators (full dataset)

Response status								
	Quit Dayn=2991	Day 7n=2341	Day 14n=2173	Day 21n=2092	Day 28n=2021	Day 35n=1987	Day 42n=1937	Day 72n=1808
	aOR (95% CI)	aOR (95% CI)	aOR (95% CI)	aOR (95% CI)	aOR (95% CI)	aOR (95% CI)	aOR (95% CI)	aOR (95% CI)
Age	1.00 (1.00 to 1.01)	1.01 (1.00 to 1.02)	1.01 (1.00 to 1.02)	1.01 (1.00 to 1.02)	1.01 (1.00 to 1.02)	1.00 (0.99 to 1.02)	1.01 (1.00 to 1.03)	1.00 (0.99 to 1.01)
Gender (ref: female)								
Male	0.80 (0.65 to 0.99)	0.88 (0.69 to 1.12)	0.69 (0.53 to 0.89)	0.91 (0.68 to 1.20)	1.05 (0.77 to 1.44)	1.12 (0.80 to 1.58)	0.96 (0.65 to 1.41)	0.80 (0.57 to 1.12)
Other	0.28 (0.04 to 2.10)	0.39 (0.05 to 3.03)	*	0.64 (0.08 to 5.05)	*	*	*	†
Region (ref: South)								
Northeast	1.07 (0.80 to 1.42)	1.15 (0.84 to 1.57)	0.98 (0.70 to 1.38)	1.09 (0.75 to 1.58)	0.94 (0.61 to 1.46)	1.30 (0.83 to 2.03)	0.69 (0.39 to 1.23)	0.89 (0.58 to 1.36)
Midwest	0.82 (0.57 to 1.17)	0.93 (0.63 to 1.37)	1.10 (0.74 to 1.64)	0.98 (0.62 to 1.54)	1.36 (0.85 to 2.19)	0.95 (0.53 to 1.69)	1.30 (0.73 to 2.30)	0.37 (0.18 to 0.74)
West	0.98 (0.75 to 1.29)	1.06 (0.79 to 1.43)	0.72 (0.51 to 1.03)	0.92 (0.64 to 1.33)	1.15 (0.77 to 1.71)	1.12 (0.72 to 1.74)	1.00 (0.61 to 1.64)	0.69 (0.44 to 1.07)
Puerto Rico and Virgin Islands	0.86 (0.51 to 1.45)	0.57 (0.29 to 1.11)	0.93 (0.51 to 1.72)	0.75 (0.36 to 1.54)	0.93 (0.42 to 2.06)	0.81 (0.31 to 2.15)	1.13 (0.48 to 2.68)	0.88 (0.40 to 1.92)
Smoking frequency (ref: non-daily)								
Daily	1.49 (1.03 to 2.17)	1.02 (0.71 to 1.47)	0.98 (0.64 to 1.51)	0.83 (0.54 to 1.28)	0.89 (0.55 to 1.45)	0.90 (0.53 to 1.53)	1.27 (0.65 to 2.48)	0.58 (0.36 to 0.93)
Cigarettes smoked per day (ref: light)								
Moderate	0.78 (0.61 to 1.00)	0.62 (0.46 to 0.82)	0.79 (0.57 to 1.09)	0.87 (0.62 to 1.21)	0.69 (0.47 to 1.01)	0.71 (0.46 to 1.10)	0.64 (0.41 to 1.01)	0.76 (0.50 to 1.16)
Heavy	0.27 (0.18 to 0.40)	0.26 (0.17 to 0.39)	0.28 (0.17 to 0.48)	0.30 (0.16 to 0.54)	0.29 (0.17 to 0.51)	0.34 (0.19 to 0.62)	0.32 (0.16 to 0.65)	0.34 (0.18 to 0.65)
Prequit time	0.91 (0.89 to 0.93)	0.93 (0.91 to 0.95)	0.91 (0.89 to 0.94)	0.92 (0.90 to 0.95)	0.92 (0.89 to 0.95)	0.92 (0.88 to 0.95)	0.92 (0.88 to 0.96)	0.90 (0.87 to 0.94)
No of quit attempts (ref: 1)								
2+	1.58 (0.97 to 2.57)	3.03 (1.89 to 4.84)	2.91 (1.72 to 4.92)	2.90 (1.66 to 5.04)	3.12 (1.70 to 5.74)	3.00 (1.55 to 5.78)	3.41 (1.67 to 6.98)	3.39 (1.75 to 6.59)
Intervention targeting (ref: Latinos, SFTXT)								
Latinos, SFTXTesp	1.35 (1.08 to 1.70)	1.17 (0.91 to 1.49)	1.28 (0.97 to 1.68)	1.33 (0.98 to 1.80)	1.05 (0.76 to 1.45)	0.95 (0.66 to 1.35)	1.84 (1.21 to 2.82)	1.82 (1.26 to 2.62)

Smoking status								
	Quit Dayn=2991	Day 7n=2991	Day 14n=2991	Day 21n=2991	Day 28n=2991	Day 35n=2991	Day 42n=2991	Day 72n=2805
	aOR (95% CI)	aOR (95% CI)	aOR (95% CI)	aOR (95% CI)	aOR (95% CI)	aOR (95% CI)	aOR (95% CI)	aOR (95% CI)
Age	1.01 (1.00 to 1.02)	1.01 (1.00 to 1.02)	1.01 (1.00 to 1.02)	1.01 (1.00 to 1.03)	1.01 (0.99 to 1.02)	1.01 (0.99 to 1.02)	1.01 (0.99 to 1.03)	1.01 (0.99 to 1.02)
Gender (ref: female)								
Male	0.71 (0.53 to 0.95)	1.16 (0.84 to 1.61)	0.70 (0.49 to 1.00)	0.92 (0.64 to 1.33)	1.09 (0.72 to 1.65)	1.09 (0.70 to 1.70)	0.79 (0.49 to 1.27)	0.73 (0.46 to 1.14)
Other	0.44 (0.06 to 3.36)	0.77 (0.10 to 5.97)	*	0.83 (0.11 to 6.43)	*	*	*	†
Region (ref: South)								
Northeast	1.08 (0.74 to 1.58)	1.53 (1.02 to 2.31)	0.94 (0.59 to 1.49)	1.26 (0.80 to 1.99)	1.52 (0.88 to 2.61)	1.40 (0.77 to 2.54)	0.78 (0.39 to 1.54)	0.83 (0.45 to 1.51)
Midwest	0.96 (0.60 to 1.53)	1.26 (0.76 to 2.08)	1.40 (0.85 to 2.29)	1.33 (0.78 to 2.26)	1.75 (0.95 to 3.23)	1.62 (0.84 to 3.15)	0.95 (0.45 to 2.04)	0.42 (0.16 to 1.08)
West	1.13 (0.80 to 1.61)	0.86 (0.56 to 1.34)	0.80 (0.51 to 1.26)	0.83 (0.52 to 1.34)	1.30 (0.77 to 2.21)	1.24 (0.70 to 2.19)	1.04 (0.58 to 1.86)	0.87 (0.50 to 1.51)
Puerto Rico and Virgin Islands	1.16 (0.60 to 2.24)	0.72 (0.28 to 1.86)	0.97 (0.42 to 2.22)	0.14 (0.02 to 1.02)	0.28 (0.04 to 2.07)	1.03 (0.30 to 3.53)	1.33 (0.49 to 3.58)	0.93 (0.35 to 2.47)
Smoking frequency (ref: non-daily)								
Daily	1.13 (0.72 to 1.77)	1.23 (0.73 to 2.07)	1.20 (0.68 to 2.12)	0.75 (0.45 to 1.27)	0.89 (0.49 to 1.59)	0.79 (0.42 to 1.49)	1.21 (0.56 to 2.63)	0.69 (0.36 to 1.30)
Cigarettes smoked per day (ref: light)								
Moderate	0.77 (0.55 to 1.08)	0.59 (0.41 to 0.85)	0.65 (0.43 to 1.00)	0.72 (0.47 to 1.11)	0.61 (0.37 to 1.00)	0.66 (0.38 to 1.15)	0.64 (0.36 to 1.13)	1.05 (0.61 to 1.81)
Heavy	0.31 (0.19 to 0.50)	0.21 (0.12 to 0.36)	0.15 (0.07 to 0.32)	0.29 (0.16 to 0.54)	0.21 (0.11 to 0.43)	0.33 (0.16 to 0.68)	0.37 (0.17 to 0.80)	0.38 (0.17 to 0.88)
Prequit time	0.90 (0.87 to 0.93)	0.95 (0.92 to 0.98)	0.95 (0.91 to 0.98)	0.93 (0.90 to 0.97)	0.95 (0.91 to 0.99)	0.93 (0.89 to 0.98)	0.93 (0.88 to 0.98)	0.90 (0.85 to 0.95)
No of quit attempts (ref: 1)								
2+	1.09 (0.54 to 2.23)	2.40 (1.30 to 4.40)	2.27 (1.18 to 4.33)	3.10 (1.64 to 5.86)	2.22 (1.02 to 4.82)	2.40 (1.06 to 5.46)	1.77 (0.68 to 4.60)	1.80 (0.69 to 4.68)
Intervention targeting (ref: Latinos, SFTXT)								
Latinos, SFTXTesp	0.95 (0.71 to 1.28)	0.80 (0.57 to 1.11)	0.74 (0.52 to 1.05)	1.04 (0.72 to 1.51)	0.69 (0.45 to 1.05)	0.66 (0.42 to 1.04)	1.12 (0.68 to 1.82)	1.31 (0.82 to 2.10)

Logistic regression modeledmodelled the probability of 1=responder in response status models and 1=nonsmoker in smoking status models.

Bolded cells represent significant results.

* aOR was undefined.

† All users who selected ‘non-binary’ or ‘prefer not to say’ had less than 72 days between quit day and end of study date and were excluded from day 72 analyses.

aOR, adjusted OR

**Table 4:** Correlates of engagement and abstinence outcomes among SmokefreeTXT en Español (SFTXTesp) and SmokefreeTXT (SFTXT) Latino and White intervention initiators (full dataset)

Response status								
	Quit Dayn=9934	Day 7n=7461	Day 14n=6721	Day 21n=6404	Day 28n=6131	Day 35n=5966	Day 42n=5777	Day 72n=5315
	aOR (95% CI)	aOR (95% CI)	aOR (95% CI)	aOR (95% CI)	aOR (95% CI)	aOR (95% CI)	aOR (95% CI)	aOR (95% CI)
Age	1.02 (1.01 to 1.02)	1.02 (1.01 to 1.02)	1.01 (1.01 to 1.02)	1.02 (1.02 to 1.03)	1.02 (1.02 to 1.03)	1.02 (1.02 to 1.03)	1.03 (1.02 to 1.03)	1.02 (1.02 to 1.03)
Gender (ref: female)								
Male	0.74 (0.66 to 0.82)	0.74 (0.66 to 0.83)	0.76 (0.67 to 0.87)	0.76 (0.66 to 0.88)	0.82 (0.71 to 0.96)	0.91 (0.78 to 1.07)	0.82 (0.68 to 0.99)	0.75 (0.63 to 0.88)
Other	0.22 (0.05 to 0.91)	0.27 (0.06 to 1.15)	0.39 (0.09 to 1.65)	0.51 (0.12 to 2.21)	0.26 (0.04 to 1.99)	*	*	†
Region (ref: South)								
Northeast	0.89 (0.77 to 1.03)	1.12 (0.96 to 1.31)	1.11 (0.94 to 1.32)	1.06 (0.88 to 1.28)	1.07 (0.87 to 1.31)	1.08 (0.88 to 1.34)	1.10 (0.86 to 1.41)	1.00 (0.80 to 1.25)
Midwest	1.11 (0.97 to 1.27)	1.16 (1.00 to 1.34)	1.14 (0.97 to 1.34)	1.25 (1.05 to 1.48)	1.34 (1.11 to 1.61)	1.27 (1.05 to 1.55)	1.48 (1.18 to 1.85)	1.11 (0.90 to 1.36)
West	1.09 (0.94 to 1.25)	1.21 (1.03 to 1.41)	0.95 (0.79 to 1.13)	1.10 (0.91 to 1.33)	1.22 (1.00 to 1.49)	1.20 (0.97 to 1.48)	1.36 (1.06 to 1.73)	1.00 (0.80 to 1.26)
Puerto Rico and Virgin Islands	0.85 (0.51 to 1.41)	0.57 (0.30 to 1.08)	0.99 (0.55 to 1.79)	0.77 (0.38 to 1.56)	0.91 (0.42 to 1.98)	0.77 (0.30 to 1.98)	1.30 (0.56 to 3.01)	1.06 (0.50 to 2.26)
Smoking frequency (ref: non-daily)								
Daily	1.06 (0.88 to 1.28)	1.00 (0.82 to 1.23)	1.13 (0.90 to 1.43)	0.94 (0.74 to 1.19)	0.92 (0.71 to 1.19)	0.85 (0.65 to 1.10)	1.03 (0.75 to 1.41)	0.70 (0.54 to 0.92)
Cigarettes smoked per day (ref: light)								
Moderate	0.90 (0.79 to 1.02)	0.79 (0.69 to 0.91)	0.88 (0.76 to 1.04)	0.86 (0.73 to 1.02)	0.76 (0.64 to 0.92)	0.82 (0.68 to 0.99)	0.71 (0.58 to 0.88)	0.76 (0.62 to 0.93)
Heavy	0.48 (0.42 to 0.56)	0.49 (0.42 to 0.58)	0.51 (0.42 to 0.61)	0.49 (0.40 to 0.60)	0.50 (0.41 to 0.61)	0.50 (0.40 to 0.62)	0.50 (0.39 to 0.64)	0.52 (0.41 to 0.66)
Prequit time	0.89 (0.88 to 0.90)	0.90 (0.89 to 0.91)	0.89 (0.88 to 0.90)	0.90 (0.88 to 0.91)	0.89 (0.87 to 0.90)	0.89 (0.88 to 0.91)	0.89 (0.87 to 0.91)	0.90 (0.88 to 0.92)
No of quit attempts (ref: 1)								
2+	1.65 (1.27 to 2.14)	2.46 (1.87 to 3.24)	2.39 (1.76 to 3.24)	2.36 (1.71 to 3.26)	2.29 (1.61 to 3.26)	2.49 (1.74 to 3.57)	2.31 (1.53 to 3.50)	2.07 (1.39 to 3.08)
Race and ethnicity (ref: Whites, SFTXT)								
Latinos, SFTXTesp	0.68 (0.58 to 0.79)	0.52 (0.43 to 0.61)	0.57 (0.47 to 0.68)	0.56 (0.45 to 0.68)	0.49 (0.39 to 0.61)	0.38 (0.29 to 0.49)	0.60 (0.46 to 0.78)	0.64 (0.51 to 0.81)
Latinos, SFTXT	0.45 (0.38 to 0.54)	0.41 (0.34 to 0.49)	0.41 (0.33 to 0.51)	0.38 (0.30 to 0.48)	0.42 (0.33 to 0.54)	0.38 (0.29 to 0.49)	0.30 (0.21 to 0.42)	0.33 (0.25 to 0.45)

Smoking status								
	Quit Day n=9934	Day 7 n=9934	Day 14 n=9934	Day 21 n=9934	Day 28 n=9934	Day 35 n=9934	Day 42 n=9934	Day 72 n=9236
	aOR (95% CI)	aOR (95% CI)	aOR (95% CI)	aOR (95% CI)	aOR (95% CI)	aOR (95% CI)	aOR (95% CI)	aOR (95% CI)
Age	1.02 (1.02 to 1.02)	1.03 (1.02 to 1.03)	1.02 (1.02 to 1.03)	1.03 (1.02 to 1.03)	1.03 (1.02 to 1.03)	1.03 (1.02 to 1.03)	1.03 (1.02 to 1.04)	1.03 (1.02 to 1.03)
Gender (ref: female)								
Male	0.76 (0.67 to 0.87)	0.75 (0.65 to 0.86)	0.77 (0.66 to 0.89)	0.82 (0.70 to 0.96)	0.82 (0.69 to 0.97)	0.88 (0.75 to 1.05)	0.85 (0.70 to 1.04)	0.79 (0.65 to 0.96)
Other	0.38 (0.09 to 1.59)	0.46 (0.11 to 1.94)	0.27 (0.04 to 1.97)	0.67 (0.16 to 2.86)	0.36 (0.05 to 2.65)	*	*	†
Region (ref: South)								
Northeast	0.94 (0.80 to 1.11)	1.37 (1.15 to 1.64)	1.22 (1.01 to 1.47)	1.16 (0.95 to 1.42)	1.17 (0.94 to 1.45)	1.24 (0.99 to 1.55)	1.28 (0.98 to 1.66)	1.05 (0.81 to 1.35)
Midwest	1.16 (1.00 to 1.35)	1.30 (1.10 to 1.54)	1.27 (1.06 to 1.51)	1.38 (1.15 to 1.65)	1.41 (1.16 to 1.72)	1.43 (1.17 to 1.76)	1.53 (1.21 to 1.95)	1.26 (1.00 to 1.59)
West	1.10 (0.93 to 1.29)	1.19 (0.99 to 1.43)	1.05 (0.86 to 1.29)	1.08 (0.88 to 1.32)	1.24 (1.00 to 1.55)	1.29 (1.03 to 1.62)	1.48 (1.14 to 1.92)	1.19 (0.93 to 1.54)
Puerto Rico and Virgin Islands	1.11 (0.58 to 2.10)	0.69 (0.27 to 1.74)	1.07 (0.48 to 2.39)	0.14 (0.02 to 1.04)	0.23 (0.03 to 1.68)	0.91 (0.28 to 3.00)	1.66 (0.64 to 4.32)	1.21 (0.47 to 3.11)
Smoking frequency (ref: non-daily)								
Daily	0.89 (0.72 to 1.10)	1.10 (0.87 to 1.41)	1.10 (0.85 to 1.43)	0.91 (0.71 to 1.18)	0.89 (0.68 to 1.16)	0.86 (0.65 to 1.14)	0.94 (0.68 to 1.30)	0.95 (0.69 to 1.31)
Cigarettes smoked per day (ref: light)								
Moderate	0.88 (0.76 to 1.03)	0.82 (0.70 to 0.96)	0.85 (0.71 to 1.01)	0.82 (0.69 to 0.99)	0.76 (0.63 to 0.93)	0.82 (0.67 to 1.00)	0.74 (0.59 to 0.93)	0.79 (0.63 to 1.00)
Heavy	0.47 (0.40 to 0.57)	0.52 (0.43 to 0.63)	0.49 (0.40 to 0.60)	0.49 (0.40 to 0.61)	0.52 (0.41 to 0.65)	0.52 (0.41 to 0.66)	0.51 (0.39 to 0.67)	0.56 (0.43 to 0.72)
Prequit time	0.87 (0.86 to 0.88)	0.90 (0.88 to 0.91)	0.91 (0.89 to 0.92)	0.91 (0.90 to 0.93)	0.90 (0.89 to 0.92)	0.91 (0.89 to 0.93)	0.91 (0.89 to 0.93)	0.91 (0.89 to 0.93)
No of quit attempts (ref: 1)								
2+	1.44 (1.05 to 1.99)	1.82 (1.32 to 2.50)	1.75 (1.26 to 2.44)	1.82 (1.29 to 2.56)	1.99 (1.39 to 2.85)	2.14 (1.50 to 3.06)	1.74 (1.13 to 2.68)	1.38 (0.87 to 2.20)
Race and ethnicity (ref: Whites, SFTXT)								
Latinos, SFTXTesp	0.42 (0.34 to 0.51)	0.39 (0.31 to 0.50)	0.40 (0.31 to 0.52)	0.47 (0.37 to 0.61)	0.35 (0.25 to 0.47)	0.28 (0.19 to 0.39)	0.45 (0.32 to 0.64)	0.53 (0.39 to 0.72)
Latinos, SFTXT	0.41 (0.33 to 0.51)	0.41 (0.32 to 0.52)	0.45 (0.35 to 0.57)	0.40 (0.31 to 0.53)	0.42 (0.31 to 0.55)	0.38 (0.28 to 0.51)	0.37 (0.26 to 0.53)	0.35 (0.24 to 0.50)

Logistic regression modeledmodelled the probability of 1=responder in response status models and 1=non-smoker in smoking status models.

Bolded cells represent significant results.

* aOR was undefined.

† All users who selected ‘non-binary’ or ‘prefer not to say’ had less than 72 days between quit day and end of study date and were excluded from day 72 analyses.

aOR, adjusted OR

### Abstinence

Overall point prevalence abstinence was 14.46% on quit day, 5.09% on day 42 and 5.73% on day 72 (table 2). SFTXTesp and SFTXT Latino users were equally likely to be abstinent across all assessment time points (table 3). Compared with SFTXT White users, SFTXTesp and SFTXT Latino users were less likely to be abstinent across all assessment time points (eg, aORs 0.42, 0.41 on quit day; 0.45, 0.37 on day 42; and 0.53, 0.35 on day 72) (table 4). Intervention completer (online supplemental tables 5 and 7) and complete case (online supplemental tables 8 and 10) analyses mirrored these patterns where SFTXTesp and SFTXT Latino users were less likely to be abstinent than SFTXT White users across all assessment time points. Among intervention completers, SFTXTesp Latino users were less likely to abstinent than SFTXT Latino users on quit day and days 7, 14, 28 and 35 (online supplemental table 6). Similar patterns emerged under complete-case analysis for quit day and days 14, 28 and 35 (online supplemental table 9).

## Discussion

This is the first study, to our knowledge, examining if intervention targeting improves retention, engagement and abstinence outcomes among its intended audience and versus a majority racial/ethnic group. In a real-world assessment of Smokefree.gov text messaging smoking cessation interventions, targeting improved retention and engagement, but not abstinence, among Latinos. Whites outperformed Latino users of both targeted and non-targeted interventions in engagement and abstinence rates, indicating that targeting did not reduce disparities between Latinos and Whites. Results suggest that rather than relying solely on linguistic targeting, Latino-targeting interventions should incorporate cultural adaptations of their content to improve intervention outcomes and reduce smoking cessation disparities.^30^ High cellphone penetration among Latinos presents an opportunity to deliver text messaging smoking cessation interventions to offset their limited access to traditional smoking cessation aids and resources.^4 5^ Text messaging interventions are especially necessary for hard-to-reach populations that do not own smartphones (eg, foreign-born Latinos)^31^ or have limited digital literacy skills.^32^ Importantly, text messaging interventions support behavioural change techniques (eg, action cues) and allow sufficient tailoring and interactivity to engage users.^33^ However, text messaging remains an underused intervention platform despite its simplicity, widespread use, user familiarity, 98% open rate and low-cost scalability.^33 34^

Targeting improved retention and engagement. At 69% and 60%, retention was higher among SFTXTesp and SFTXT Latino users than among SFTXT White users (56%). Results reflect similar patterns where vulnerable groups (eg, Blacks, low socioeconomic smokers) remain in Smokefree.gov text messaging interventions.^35 36^ Our retention rates were lower than those reported for general behavioural health (eg, 86% in a meta-analysis of 19 randomised trials)^10^ and targeted smoking cessation (eg, 85% at 12-week follow-up in small-scale study)^37^ interventions. For engagement, response rates were higher among SFTXTesp Latino users than SFTXT Latino users, but Latinos were less engaged than Whites throughout the intervention. Measurement heterogeneity impedes comparisons across studies.^24^ For example, previous studies used programme completion,^21^ user-initiated text messages^20 26^ and survey responses^26^ as engagement metrics. Since intervention efficacy depends on retention and engagement,^2224^,^27^ research is needed to identify user characteristics and intervention features associated with improved retention and engagement^38 39^ and to unpack the associations between retention, engagement and abstinence, especially given that retention and engagement did not translate to abstinence among Latinos (vs Whites) in our study.

Several results are worth highlighting regarding abstinence. First, by intervention end, overall abstinence among SFTXTesp and SFTXT intervention initiators was slightly lower than previously reported among SFTXT users (5.09% vs 7.2%).^21^ Among intervention completers, overall abstinence was 8.77% consistent with evidence of improved abstinence among SFTXT users who get the full treatment dose (12.9%).^21^ Consistent with literature,^21^ abstinence was attenuated at 1-month postintervention completion. Second, at 6.19% and 11.21%, abstinence rates among SFTXT hite intervention initiators and completers tracked closely those reported in the literature. Abstinence among SFTXTesp and SFTXT Latino users were roughly halved to 3.03% and 2.12% among intervention initiators and to 4.41% and 3.47% among intervention completers. Abstinence rates among SFTXTesp Latino users were lower than previously reported in pilot testing of targeted text messaging interventions for Latinos such as Quitxt (25.2%),^17^ Latino Kick Buts (30%)^37^ and Vive sin Tabaco… ¡Decídete! (40%).^20^ Third, abstinence rates were equivalent among SFTXTesp and SFTXT Latino users. Among intervention completers and those without missing data, SFTXTesp users underperformed their SFTXT counterparts on abstinence at various assessment times. Despite evidence that targeting improves intervention outcomes,^10^ SFTXTesp showed that relying on linguistic targeting only did not improve abstinence among its users compared with SFTXT White users.

Observed abstinence rates between SFTXTesp and SFTXT Latino users and between Latino and White users may suggest that linguistic targeting is insufficient to improve SFTXTesp efficacy. With cultural intervention adaptations being well received by intended populations,^18^ SFTXTesp content could emphasise Latino values (eg, familismo, respeto) and address Latino-specific factors related to smoking and cessation (eg, acculturative stress).^40^ The heterogeneity of Latino smokers presents an additional explanation for the observed abstinence outcomes. SFTXTesp Latino users were more likely to be older, male, from Puerto Rico and the Virgin Islands, and were less likely to smoke heavily (online supplemental table 11). This is particularly important given differences in smoking patterns among Latinos by sex, country of origin and acculturation,^2^ suggesting a need for tailoring to improve intervention efficacy above and beyond targeting.^10^ Lower abstinence rates among SFTXTesp (vs SFTXT) intervention completers and under a complete-case analysis could be attributed to the high percentage of abstinent Latinos SFTXTesp users who were excluded from the intervention completer and the complete-case datasets. Of those excluded from the completer dataset, 19.01% SFTXTesp Latino users (n=116 abstinent/ 610 excluded), 7.11% SFTXT Latino users (n=94/1322) and 23.54% SFTXT white users (n=1081/4591) were abstinent. Of those excluded from the complete-case dataset, 40.06% SFTXTesp Latino users (n=242 abstinent/ 604 excluded), 14% SFTXT Latino users (n=7/50) and 27.27% SFTXT White users (n=3/11) were abstinent.

Smokefree.gov interventions should allow each user to further tailor the intervention based on a unique set of outcome or behaviour related factors, which could further improve efficacy of targeted interventions.^10 15^ (CF^29^ Elements for content tailoring could include smoking frequency, intensity and prior quit attempts where Smokefree.gov interventions would allow for tailored message frequency, timing and content based on smokers’ input at baseline. Future research should explore whether a preparation phase is beneficial given that we found longer prequit times were associated with worse intervention outcomes,^9^ and solicit qualitative feedback from SFTXTesp and SFTXT Latino users who (un)successfully used the intervention on how to improve retention, engagement and abstinence.^30^ Finally, future research should re-evaluate intervention outcomes for SFTXTesp and SFTXT since both interventions are updated periodically (eg, in 2019 after data were pulled for this study).

Strengths of the study include reporting on a real-world assessment of Latino-targeting SFTXTesp text messaging smoking cessation intervention. The unique parallels between SFTXTesp and SFTXT afforded a quasi-experimental design to examine whether targeting improves intervention outcomes among its intended Latino population and when compared with Whites. Although Latinos were not randomly assigned to either SFTXTesp or SFTXT, comparisons of targeted versus non-targeted interventions have been typically limited to the target population (eg, Latinos) and have been researcher-controlled studies with incentivised participation. Limitations include unmeasured factors (eg, nicotine dependence) that could have impacted intervention outcomes. Drop-outs were users who actively texted ‘STOP’ or for whom text messages were undeliverable but did not include passive drop-out where users disengage from the intervention, potentially inflating retention. Drop-outs were considered smokers in intention-to-treat analysis. However, users could have dropped out because they had already quit smoking.^37^ Engagement and abstinence rates for each assessment time point were independent of prior or subsequent assessment points. Accordingly, we could not capture disengagement/reengagement, continued abstinence or relapses. Using response to smoking status prompts as an engagement metric could underestimate engagement among users who were not abstinent.^26^ Other factors could have been used as engagement metrics (eg, user-initiated text). Abstinence was self-reported and short-term. Users with >1 quit attempt had longer prequit time that reflected time from signup to quit day (rather than quit day reset date). SFTXTesp had a high percentage of missingness. However, we imputed missing data and ran sensitivity analyses using intervention completer and complete-case datasets.

### Conclusion

SFTXTesp, a Latino-targeting text messaging smoking cessation intervention, resulted in higher retention and engagement rates among its users than among Latino users of SFTXT, a non-targeted intervention. Linguistic targeting did not improve abstinence among Latinos or reduce disparities between Latinos and Whites in engagement and abstinence rates. Cultural adaptation and tailoring are necessary to maximise the efficacy of targeted smoking cessation interventions.

## supplementary material

10.1136/bmjph-2023-000222 online supplemental file 1

## Data Availability

Data are available on reasonable request.
