# Supplementary material for: Intervention targeting and retention, engagement and abstinence outcomes among Latino and White users of Smokefree.gov text messaging programmes: a cross-sectional study
Source: BMJ Public Health. 2023 Nov 8;1(1):e000222. doi: 10.1136/bmjph-2023-000222 (PMC10732326; doi:10.1136/bmjph-2023-000222)
Supplement: online supplemental file 1 [file bmjph-1-1-s001.pdf]

Supplementary Table 1: Exclusion criteria for SmokefreeTXT en Español (SFTXTesp) and SmokefreeTXT (SFTXT) Latino and White web enrollees

	Latinos, SFTXTesp		Latinos, SFTXT		Whites, SFTXT		Total	
	n	%	*n*	%	*n*	%	*N*	%
Total eligible web-enrolled subscribers	1774	100	3123	100	12,362	100	17259	100
First reason for exclusion								
Multiple sign-ups	148	8.34	437	13.99	2,936	23.75	3,521	20.40
Quit day before signup date	12	0.68	37	1.18	116	0.94	165	0.96
Opted out before prequit period	1	0.06	0	0.00	0	0.00	1	.01
Less than 42 days of post-quit day time prior to end of study period	51	2.87	348	11.14	892	7.22	1,291	7.48
Excluded from study	212	11.95	822	26.32	3,944	31.90	4,978	28.84
Included in study	1562	88.05	2,301	73.68	8,418	68.10	12,281	71.16

Ineligible for inclusion in the study were 33,385 SFTXT records of users (a) who signed up for the intervention via text message and were not asked the race/ethnicity questions (*n*= 20257), (b) who had missing race and/or ethnicity data (*n*= 9256), and (c) who belonged to racial/ethnic groups other than Whites and Latinos (*n*= 3872).

Supplementary Table 2: Sample characteristics by intervention targeting, race, and ethnicity (full dataset, *N*= 12,281)

	**Latinos, SFTXTesp**						**Latinos, SFTXT**						**Whites, SFTXT**					
	Total		Non-completers		Completers		Total		Non-completers		Completers		Total		Non-completers		Completers	
	N	*SS*	*N*	*SS*	*N*	*SS*	*N*	*SS*	*N*	*SS*	*N*	*SS*	*N*	*SS*	*N*	*SS*	*N*	*SS*
**Age,** M (SD)	1562	38.26 (11.46)	610	37.83 (11.07)	952	38.54 (11.71)	2301	35.97 (16.65)	1322	35.38 (17.15)	979	36.77 (15.85)	8418	37.92 (13.53)	4591	36.34 (13.35)	3827	39.81 (13.50)
5% Trimmed M (SD)	1406	37.84 (11.04)	549	37.46 (10.91)	857	38.09 (11.15)	2071	34.23 (15.39)	1190	33.48 (15.69)	881	35.24 (14.68)	7576	37.30 (13.55)	4132	35.56 (13.20)	3444	39.41 (13.80)
Range	1562	14-97	610	17-75	952	14-97	2301	17-99	1322	17-99	979	18-99	8418	16-99	4591	17-99	3827	16-99
Median (IQR)	1562	37 (15)	610	36 (15)	952	37 (16)	2301	31 (21)	1322	30 (20)	979	33 (21)	8418	36 (22)	4591	34 (21)	3827	38 (23)
**Gender, %**																		
Female	773	49.51	307	50.31	466	49.00	1202	52.24	701	53.03	501	51.17	5195	61.71	2769	60.31	2426	63.39
Male	789	50.49	303	49.69	486	51.00	1031	44.81	578	43.72	453	46.27	3200	38.01	1807	39.36	1393	36.40
Other	-	-	-	-	-	-	68	2.96	43	3.25	25	2.55	23	0.27	15	0.33	8	0.21
**Region, %**																		
Northeast	280	17.90	101	16.54	179	18.77	470	20.44	261	19.74	209	21.39	1613	19.17	851	18.54	762	19.92
Midwest	181	11.61	50	8.12	132	13.85	368	15.98	236	17.85	132	13.44	2176	25.85	1168	25.44	1008	26.34
South	647	41.40	263	43.06	384	40.33	867	37.66	488	36.91	379	38.67	3228	38.35	1838	40.03	1390	36.33
West	350	22.43	155	25.41	195	20.53	577	25.10	330	24.96	247	25.28	1400	16.63	733	15.97	667	17.42
Puerto Rico & Virgin Islands	104	6.66	42	6.87	62	6.53	19	0.83	7	0.53	12	1.23	1	0.01	1	0.02	0	0.0
**Smoking frequency, %**																		
Nondaily	170	10.85	72	11.80	98	10.24	225	9.78	122	9.23	103	10.52	639	7.59	340	7.41	299	7.81
Daily	1393	89.15	538	88.20	855	89.76	2076	90.22	1200	90.77	876	89.48	7779	92.41	4251	92.59	3528	92.19
**Cigarettes smoked per day,** %																		
Light	602	38.56	247	40.50	355	37.32	508	22.08	242	18.31	266	27.17	1922	22.83	995	21.67	927	24.21
Moderate	681	43.60	257	42.11	424	44.55	484	21.05	248	18.76	236	24.14	3308	39.30	1744	37.99	1564	40.87
Heavy	279	17.84	106	17.39	173	18.13	1309	56.88	832	62.93	477	48.69	3188	37.87	1852	40.34	1336	34.92
**Time from signup to quit day,** M (SD)	1387	6.52 (28.12)	435	3.98 (10.90)	952	7.69 (33.06)	1604	6.00 (17.06)	625	3.46 (11.62)	979	7.62 (19.59)	6943	5.59 (18.63)	3116	3.38 (7.62)	3827	7.39 (23.98)
5% Trimmed M (SD)	1248	3.71 (5.25)	392	2.53 (4.75)	857	4.24 (5.39)	1444	4.45 (5.98)	563	2.23 (4.91)	881	5.87 (6.20)	6249	3.79 (5.66)	2804	2.65 (5.03)	3444	4.71 (5.98)
Range	1387	0 – 615	435	0 – 125	952	0 – 615	1604	0 – 423	625	0 – 181	979	0 – 423	6943	0 – 412	3116	0 – 218	3827	0 – 412
Median (IQR)	1387	1 (7)	435	1 (4)	952	2 (8)	1604	1 (11)	625	0 (3)	979	4 (14)	6943	1 (8)	3116	1 (4)	3827	2 (11)

**Time from signup to dropout**, M (SD)	610	12.17 (36.28)	610	12.17 (36.28)	-	-	1322	5.52 (12.86)	1322	5.52 (12.86)	-	-	4591	8.85 (12.42)	4591	8.85 (12.42)	-	-
5% Trimmed M (SD)	549	9.05 (12.31)	549	9.05 (12.31)	-	-	1190	3.53 (8.15)	1190	3.53 (8.15)	-	-	4132	7.33 (11.00)	4132	7.33 (11.00)	-	-
Range	610	0 – 823	610	0 – 823	-	-	1322	0 – 182	1322	0 – 182	-	-	4591	0 – 236	4591	0 – 236	-	-
Median (IQR)	610	4 (16)	610	4 (16)	-	-	1322	1 (5)	1322	1 (5)	-	-	4591	4 (12)	4591	4 (12)	-	-
**Time from quit day to dropout**, M (SD)	435	10.40 (11.13)	435	10.40 (11.13)	-	-	625	6.64 (10.15)	625	6.64 (10.15)	-	-	3116	8.73 (10.55)	3116	8.73 (10.55)	-	-
5% Trimmed M (SD)	392	9.47 (11.54)	392	9.47 (11.54)	-	-	563	5.28 (9.80)	563	5.28 (9.80)	-	-	2804	7.60 (10.59)	2804	7.60 (10.59)	-	-
Range	435	0 – 42	435	0 – 42	-	-	625	0 – 42	625	0 – 42	-	-	3116	0 – 42	3116	0 – 42	-	-
Median (IQR)	435	7 (16)	435	7 (16)	-	-	625	2 (9)	625	2 (9)	-	-	3116	4 (11)	3116	4 (11)	-	-
**Prequit time**, M (SD)	1562	3.80 (4.87)	610	2.67 (4.21)	952	4.52 (5.12)	2301	3.58 (5.17)	1322	1.80 (3.67)	979	5.98 (5.89)	8418	3.71 (5.11)	4591	2.67 (4.31)	3827	4.94 (5.67)
5% Trimmed M (SD)	1406	3.44 (5.13)	549	2.19 (4.44)	857	4.24 (5.39)	2071	3.20 (5.45)	1190	1.22 (3.87)	881	5.87 (6.20)	7576	3.34 (5.38)	4132	2.19 (4.55)	3444	4.71 (5.98)
Range	1562	0 – 14	610	0 – 14	952	0 – 14	2301	0 – 14	1322	0 – 14	979	0 – 14	8418	0 – 14	4591	0 – 14	3827	0 – 14
Median (IQR)	1562	1 (7)	610	1 (3)	952	2 (8)	2301	1 (6)	1322	0 (1)	979	4 (14)	8418	1 (6)	4591	1 (3)	3827	2 (11)
**Time of dropout**, %																		
Before quit day	175	28.69	175	28.69	-	-	697	52.72	697	52.72	-	-	1475	32.13	1475	32.13	-	-
On or after quit day	435	71.31	435	71.31	-	-	625	47.28	625	47.28	-	-	3116	67.87	3116	67.87	-	-
**Number of quit attempts,** %																		
1	1482	94.88	572	93.77	910	95.59	2209	96.00	1278	96.67	931	95.10	8120	96.46	4437	96.65	3683	96.24
2+	80	5.12	38	6.23	42	4.41	92	4.00	44	3.33	48	4.90	298	3.54	154	3.35	144	3.76

Ns represent 1/20th of a person rounded to the nearest whole number. All summary statistics were pooled across 20 imputations.
SS: summary statistic, M: mean, SD: standard deviation, IQR: interquartile range.
All time variables (i.e., time from signup date to quit day, time from signup to dropout, time from quit day to dropout, and prequit time) are reported in days.

Supplementary Table 3. Sample characteristics by intervention targeting, race, and ethnicity (complete case dataset, *N*= 11,616)

	**Latinos, SFTXTesp**						**Latinos, SFTXT**						**Whites, SFTXT**					
	Total		Non-completers		Completers		Total		Non-completers		Completers		Total		Non-completers		Completers	
	N	*SS*	*N*	*SS*	*N*	*SS*	*N*	*SS*	*N*	*SS*	*N*	*SS*	*N*	*SS*	*N*	*SS*	*N*	*SS*
**Age,** M (SD)	958	38.27 (11.59)	340	37.99 (10.95)	618	38.43 (11.94)	2251	35.91 (16.40)	1288	35.25 (16.80)	963	36.79 (15.82)	8407	37.92 (13.53)	4587	36.34 (13.35)	3820	39.82 (13.50)
5% Trimmed M (SD)	862	37.84 (11.00)	306	37.72 (10.83)	556	37.93 (11.33)	2025	34.18 (15.19)	1158	33.37 (15.41)	865	35.25 (14.63)	7565	37.30 (13.56)	4127	35.55 (13.20)	3438	39.42 (13.80)
Range	958	17-97	340	17-75	618	17-97	2251	17-99	1288	17-99	963	18-99	8407	16-99	4587	17-99	3820	16-99
Median (IQR)	958	37 (15)	340	37 (14)	618	37 (15)	2251	31 (20)	1288	30 (20)	963	33 (21)	8407	36 (22)	4587	34 (21)	3820	38 (23)
**Gender, %**																		
Female	489	51.04	177	52.06	312	50.49	1171	52.02	677	52.56	494	51.30	5190	61.73	2768	60.34	2422	63.40
Male	469	48.96	163	47.94	306	49.51	1012	44.96	568	44.10	444	46.11	3194	37.99	1804	39.33	1390	36.39
Other	-	-	-	-	-	-	68	3.02	43	3.34	25	2.60	23	0.27	15	0.33	8	0.21
**Region, %**																		
Northeast	172	17.95	56	16.47	116	18.77	461	20.48	254	19.72	207	21.50	1612	19.17	851	18.55	761	19.92
Midwest	121	12.63	24	7.06	97	15.70	355	15.77	228	17.70	127	13.19	2173	25.85	1166	25.42	1007	26.36
South	379	39.56	145	42.65	234	37.86	849	37.72	477	37.03	372	38.63	3224	38.35	1837	40.05	1387	36.31
West	216	22.55	91	26.76	125	20.23	567	25.19	322	25.00	245	25.44	1397	16.62	732	15.96	665	17.41
Puerto Rico & Virgin Islands	70	7.31	24	7.06	46	7.44	19	0.84	7	0.54	12	1.25	1	0.01	1	0.02	0	0.00
**Smoking frequency, %**																		
Nondaily	111	11.59	49	14.41	62	10.03	221	9.82	119	9.24	102	10.59	639	7.60	340	7.41	299	7.83
Daily	847	88.41	291	85.59	556	89.97	2030	90.18	1169	90.76	861	89.41	7768	92.40	4247	92.59	3521	92.17
**Cigarettes smoked per day smoked, %**																		
Light	375	39.14	142	41.76	233	37.70	507	22.52	242	18.79	265	27.52	1920	22.84	995	21.69	925	24.21
Moderate	418	43.63	138	40.59	280	45.31	481	21.37	246	19.10	235	24.40	3306	39.32	1744	38.02	1562	40.89
Heavy	165	17.22	60	17.65	105	16.99	1263	56.11	800	62.11	463	48.08	3181	37.84	1848	40.29	1333	34.90
**Time from signup date to quit day,** M (SD)	855	7.39 (34.39)	237	3.97 (11.42)	618	8.70 (39.75)	1577	5.99 (17.19)	614	3.50 (11.72)	963	7.57 (19.75)	6935	5.59 (18.64)	3115	3.38 (7.62)	3820	7.38 (24.00)
5% Trimmed M (SD)	769	4.03 (5.36)	213	2.62 (4.70)	556	4.58 (5.50)	1419	4.41 (5.96)	552	2.25 (4.92)	865	5.78 (6.20)	6241	3.78 (5.66)	2803	2.66 (5.04)	3438	4.71 (5.98)
Range	855	0 – 615	237	0 – 125	618	0 – 615	1577	0 – 423	614	0 – 181	963	0 – 423	6935	0 – 412	3115	0 – 218	3820	0 – 412
Median (IQR)	855	2 (8)	237	1 (4)	618	2 (10)	1577	1 (10)	614	0 (3)	963	3 (14)	6935	1 (8)	3115	1 (4)	3820	2 (11)

**Time from signup to dropout,** M (SD)	340	13.04 (46.65)	340	13.04 (46.65)	--	--	1288	5.60 (12.98)	1288	5.60 (12.98)	--	--	4587	8.85 (12.43)	4587	8.85 (12.43)	--	--
5% Trimmed M (SD)	306	8.91 (12.49)	306	8.91 (12.49)	--	--	1158	3.58 (8.20)	1158	3.58 (8.20)	--	--	4127	7.33 (11.01)	4127	7.33 (11.01)	--	--
Range	340	0-823	340	0-823	--	--	1288	0-182	1288	0-182	--	--	4587	0-236	4587	0-236	--	--
Median (IQR)	340	4 (16)	340	4 (16)	--	--	1288	1 (5)	1288	1 (5)	--	--	4587	4 (12)	4587	4 (12)	--	--
**Time from quit day to dropout,** M (SD)	237	10.23 (11.52)	237	10.23 (11.52)	--	--	614	6.68 (10.17)	614	6.68 (10.17)	--	--	3115	8.73 (10.55)	3115	8.73 (10.55)	--	--
5% Trimmed M (SD)	213	9.25 (12.04)	213	9.25 (12.04)	--	--	552	5.31 (9.81)	552	5.31 (9.81)	--	--	2803	7.60 (10.60)	2803	7.60 (10.60)	--	--
Range	237	0-42	237	0-42	--	--	614	0-42	614	0-42	--	--	3115	0-42	3115	0-42	--	--
Median (IQR)	237	6 (14)	237	6 (14)	--	--	614	2 (9)	614	2 (9)	--	--	3115	4 (11)	3115	4 (11)	--	--
**Prequit time,** M (SD)	958	4.11 (4.98)	340	2.81 (4.22)	618	4.82 (5.22)	2251	3.57 (5.17)	1288	1.81 (3.69)	963	5.91 (5.88)	8407	3.70 (5.10)	4587	2.68 (4.32)	3820	4.94 (5.67)
5% Trimmed M (SD)	862	3.79 (5.25)	306	2.36 (4.33)	556	4.58 (5.50)	2025	3.18 (5.45)	1158	1.23 (3.89)	865	5.78 (6.20)	7565	3.34 (5.38)	4127	2.19 (4.55)	3438	4.71 (5.98)
Range	958	0-14	340	0-14	618	0-14	2251	0-14	1288	0-14	963	0-14	8407	0-14	4587	0-14	3820	0-14
Median (IQR)	958	1 (8)	340	1 (4)	618	2 (10)	2251	1 (6)	1288	0 (1)	963	3 (14)	8407	1 (6)	4587	1 (3)	3820	2 (11)
**Time of dropout, %**																		
Before quit day	103	30.29	103	30.29	--	--	674	52.33	674	52.33	--	--	1472	32.09	1472	32.09	--	--
On or after quit day	237	69.71	237	69.71	--	--	614	47.67	614	47.67	--	--	3115	67.91	3115	67.91	--	--
**Number of quit attempts, %**																		
1	928	96.87	324	95.29	604	97.73	2160	95.96	1244	96.58	916	95.12	8109	96.46	4433	96.64	3676	96.23
2+	30	3.13	16	4.71	14	2.27	91	4.04	44	3.42	47	4.88	298	3.54	154	3.36	144	3.77

SS: summary statistic, M: mean, SD: standard deviation, IQR: interquartile range.
All time variables (i.e., time from signup date to quit day, time from signup to dropout, time from quit day to dropout, and prequit time) are reported in days.

Supplementary Table 4: Correlates of dropping out before quit day (i.e., being an intervention non-initiator)

	Full dataset (*N*= 12,281)			Complete case dataset (*N*= 11,616)		
	aOR	LCL	UCL	aOR	LCL	UCL
Age	**0.99**	**0.98**	**0.99**	**0.98**	**0.98**	**0.98**
Gender (ref: female)						
Male	**1.24**	**1.12**	**1.37**	**1.25**	**1.14**	**1.39**
Other	1.41	0.90	2.22	1.43	0.91	2.25
Region (ref: South)						
Northeast	0.90	0.79	1.03	0.88	0.76	1.00
Midwest	0.88	0.78	1.00	**0.87**	**0.76**	**0.99**
West	0.87	0.76	1.00	0.87	0.76	1.00
Puerto Rico & Virgin Islands	**0.34**	**0.15**	**0.80**	**0.33**	**0.13**	**0.85**
Smoking Frequency (ref: nondaily)						
Daily	**0.74**	**0.61**	**0.88**	**0.70**	**0.58**	**0.84**
Cigarettes smoked per day (ref: light)						
Moderate	1.15	0.99	1.34	1.16	0.99	1.35
Heavy	**2.56**	**2.22**	**2.94**	**2.63**	**2.28**	**3.03**
Prequit time	**0.87**	**0.86**	**0.88**	**0.86**	**0.85**	**0.88**
Number of quit attempts (ref: 1)						
2+	**1.32**	**1.01**	**1.73**	**1.38**	**1.05**	**1.82**
Race and ethnicity (ref: Whites, SFTXT)						
Latinos, SFTXTesp	**0.73**	**0.61**	**0.88**	**0.73**	**0.59**	**0.92**
Latinos, SFTXT	**1.74**	**1.55**	**1.95**	**1.70**	**1.51**	**1.91**

aOR: adjusted odds ratio, LCL: lower confidence level, UCL: upper confidence level.
Bolded cells represent significant results.

Supplementary Table 5: Response rates and point-prevalence abstinence among SmokefreeTXT en Español (SFTXTesp) and SmokefreeTXT (SFTXT) intervention initiators who completed the intervention (completers dataset, *N*= 5,758)

	Did not respond, *n*	Responded		Response rate denominator, *n*	Response rate, %	Abstinence rate denominator, *n*	Abstinence, %
		Abstinent, *n*	Not Abstinent, *n*				
**Quit day**							
Latinos, SFTXTesp	802	65	85	952	15.76	952	6.83
Latinos, SFTXT	869	74	36	979	11.24	979	7.56
Whites, SFTXT	2862	701	264	3827	25.22	3827	18.32
Overall	4533	840	385	5758	21.27	5758	14.59
**Day 7**							
Latinos, SFTXTesp	806	49	97	952	15.34	952	5.15
Latinos, SFTXT	862	65	52	979	11.95	979	6.64
Whites, SFTXT	2715	706	406	3827	29.06	3827	18.45
Overall	4383	820	555	5758	23.88	5758	14.24
**Day 14**							
Latinos, SFTXTesp	816	60	76	952	14.29	952	6.30
Latinos, SFTXT	880	66	33	979	10.11	979	6.74
Whites, SFTXT	2880	685	262	3827	24.75	3827	17.90
Overall	4576	811	371	5758	20.53	5758	14.08
**Day 21**							
Latinos, SFTXTesp	825	63	64	952	13.34	952	6.62
Latinos, SFTXT	905	53	21	979	7.56	979	5.41
Whites, SFTXT	2979	645	203	3827	22.16	3827	16.85
Overall	4709	761	288	5758	18.22	5758	13.22
**Day 28**							
Latinos, SFTXTesp	852	40	60	952	10.50	952	4.20
Latinos, SFTXT	906	49	24	979	7.46	979	5.01
Whites, SFTXT	3087	590	150	3827	19.34	3827	15.42
Overall	4845	679	234	5758	15.86	5758	11.79
**Day 35**							
Latinos, SFTXTesp	875	33	44	952	8.09	952	3.47
Latinos, SFTXT	914	47	18	979	6.64	979	4.80
Whites, SFTXT	3130	574	123	3827	18.21	3827	15.00
Overall	4919	654	185	5758	14.57	5758	11.36

**Day 42**							
Latinos, SFTXTesp	869	42	41	952	8.72	952	4.41
Latinos, SFTXT	942	34	3	979	3.78	979	3.47
Whites, SFTXT	3316	429	82	3827	13.35	3827	11.21
Overall	5127	505	126	5758	10.96	5758	8.77
**Day 72**							
Latinos, SFTXTesp	819	52	66	937	12.59	938	5.54
Latinos, SFTXT	819	33	19	871	5.97	875	3.77
Whites, SFTXT	2886	444	177	3507	17.71	3534	12.56
Overall	4524	529	262	5315	14.88	5347	9.89

Supplementary Table 6: Correlates of engagement and abstinence outcomes among SmokefreeTXT en Español (SFTXTesp) and SmokefreeTXT (SFTXT) Latino intervention initiators who completed the intervention (completers dataset)

	Response status							
	**Quit Day** *n*= 1931	**Day 7** *n*= 1931	**Day 14** *n*= 1931	**Day 21** *n*= 1931	**Day 28** *n*= 1931	**Day 35** *n*= 1931	**Day 42** *n*= 1931	**Day 72** *n*= 1808
	aOR (95% CI)	aOR (95% CI)	aOR (95% CI)	aOR (95% CI)	aOR (95% CI)	aOR (95% CI)	aOR (95% CI)	aOR (95% CI)
Age	1.00 (0.99-1.01)	1.00 (0.99-1.01)	1.00 (0.99-1.02)	1.01 (0.99-1.02)	1.00 (0.99-1.02)	1.00 (0.99-1.01)	1.01 (1.00-1.03)	1.00 (0.99-1.01)
Gender (ref: female)								
Male	0.87 (0.66-1.15)	0.95 (0.72-1.26)	**0.69 (0.52-0.93)**	0.95 (0.70-1.30)	1.02 (0.73-1.41)	1.15 (0.81-1.64)	0.96 (0.65-1.41)	0.80 (0.57-1.12)
Other	-[a]	0.51 (0.06-3.97)	-[a]	0.76 (0.10-6.00)	-[a]	-[a]	-[a]	-[b]
Region (ref: South)								
Northeast	1.00 (0.69-1.44)	1.17 (0.82-1.68)	0.91 (0.62-1.33)	1.09 0.73-1.64)	0.91 (0.58-1.45)	1.27 (0.80-2.01)	0.69 (0.39-1.23)	0.89 (0.58-1.36)
Midwest	0.80 (0.51-1.27)	0.88 (0.55-1.39)	1.21 (0.79-1.87)	0.90 (0.54-1.50)	1.42 (0.87-2.33)	0.96 (0.53-1.73)	1.30 (0.74-2.30)	**0.37 (0.18-0.74)**
West	0.85 (0.59-1.23)	1.05 (0.73-1.49)	**0.56 (0.37-0.85)**	0.91 (0.61-1.36)	1.11 (0.73-1.69)	1.06 (0.67-1.68)	1.01 (0.61-1.65)	0.69 (0.44-1.07)
Puerto Rico & Virgin Islands	0.94 (0.47-1.86)	0.55 (0.24-1.27)	0.88 (0.43-1.79)	0.88 (0.41-1.90)	1.09 (0.48-2.46)	0.86 (0.32-2.27)	1.13 (0.48-2.67)	0.88 (0.40-1.92)
Smoking frequency (ref: nondaily)								
Daily	1.32 (0.82-2.13)	1.15 (0.74-1.77)	1.02 (0.62-1.67)	0.77 (0.48-1.23)	0.80 (0.49-1.31)	0.87 (0.51-1.49)	1.27 (0.65-2.49)	**0.58 (0.36-0.93)**
Cigarettes smoked per day (ref: light)								
Moderate	0.78 (0.56-1.10)	**0.58 (0.42-0.81)**	0.83 (0.58-1.19)	0.91 (0.63-1.31)	0.71 (0.48-1.05)	0.70 (0.44-1.12)	0.65 (0.41-1.02)	0.76 (0.50-1.16)
Heavy	**0.31 (0.19-0.52)**	**0.19 (0.12-0.32)**	**0.23 (0.11-0.45)**	**0.30 (0.16-0.57)**	**0.27 (0.14-0.49)**	**0.31 (0.17-0.57)**	**0.32 (0.16-0.65)**	**0.34 (0.18-0.65)**
Prequit time	**0.89 (0.86-0.92)**	**0.93 (0.90-0.96)**	**0.90 (0.87-0.93)**	**0.90 (0.87-0.94)**	**0.91 (0.88-0.94)**	**0.92 (0.89-0.96)**	**0.92 (0.88-0.96)**	**0.90 (0.87-0.94)**

Number of quit attempts (ref: 1)								
2+	1.60 (0.82-3.14)	**3.90 (2.27-6.69)**	**2.95 (1.60-5.42)**	**3.45 (1.88-6.33)**	**3.76 (2.02-7.00)**	**2.95 (1.50-5.81)**	**3.40 (1.66-6.95)**	**3.39 (1.75-6.59)**
Intervention targeting (ref: Latinos, SFTXT)								
Latinos, SFTXTesp	1.05 (0.78-1.40)	0.97 (0.73-1.29)	1.00 (0.74-1.37)	1.39 (1.00-1.93)	1.05 (0.75-1.48)	0.93 (0.64-1.34)	**1.83 (1.20-2.80)**	**1.82 (1.26-2.62)**
**Smoking status**								
	**Quit Day** *n*= 1931	**Day 7** *n*= 1931	**Day 14** *n*= 1931	**Day 21** *n*= 1931	**Day 28** *n*= 1931	**Day 35** *n*= 1931	**Day 42** *n*= 1931	**Day 72** *n*= 1813
	aOR (95% CI)	aOR (95% CI)	aOR (95% CI)	aOR (95% CI)	aOR (95% CI)	aOR (95% CI)	aOR (95% CI)	aOR (95% CI)
Age	1.01 (0.99-1.02)	1.00 (0.99-1.02)	1.01 (0.99-1.02)	1.01 (0.99-1.02)	1.00 (0.99-1.02)	1.00 (0.98-1.02)	1.01 (0.99-1.03)	1.00 (0.99-1.02)
Gender (ref: female)								
Male	**0.66 (0.46-0.95)**	1.11 (0.74-1.64)	0.69 (0.47-1.02)	0.93 (0.62-1.38)	1.01 (0.64-1.57)	1.03 (0.65-1.64)	0.72 (0.44-1.16)	0.66 (0.41-1.04)
Other	-[a]	1.00 (0.12-8.09)	-[a]	0.91 (0.11-7.37)	-[a]	-[a]	-[a]	-[b]
Region (ref: South)								
Northeast	1.17 (0.73-1.89)	1.52 (0.92-2.50)	0.78 (0.46-1.31)	1.21 (0.73-2.00)	1.27 (0.71-2.30)	1.31 (0.71-2.42)	0.73 (0.36-1.45)	0.76 (0.41-1.40)
Midwest	0.91 (0.49-1.68)	1.38 (0.75-2.53)	1.34 (0.77-2.33)	1.27 (0.70-2.31)	1.70 (0.88-3.28)	1.62 (0.81-3.25)	0.94 (0.43-2.02)	0.40 (0.15-1.05)
West	1.14 (0.72-1.81)	0.97 (0.57-1.65)	0.68 (0.41-1.15)	0.86 (0.51-1.45)	1.18 (0.66-2.10)	1.08 (0.59-2.01)	1.08 (0.60-1.96)	0.91 (0.52-1.60)
Puerto Rico & Virgin Islands	0.87 (0.33-2.33)	0.50 (0.11-2.14)	1.04 (0.42-2.61)	0.18 (0.02-1.31)	0.32 (0.04-2.39)	1.20 (0.34-4.18)	1.46 (0.53-3.99)	1.07 (0.39-2.91)
Smoking frequency (ref: nondaily)								
Daily	1.15 (0.64-2.05)	1.52 (0.78-2.95)	1.10 (0.58-2.08)	0.60 (0.35-1.04)	0.74 (0.40-1.36)	0.71 (0.37-1.37)	1.18 (0.54-2.59)	0.69 (0.36-1.31)
Cigarettes smoked per day (ref: light)								
Moderate	0.76 (0.49-1.18)	**0.57 (0.36-0.90)**	0.64 (0.40-1.02)	0.74 (0.46-1.22)	0.60 (0.35-1.02)	0.70 (0.39-1.25)	0.64 (0.36-1.12)	1.06 (0.61-1.84)
Heavy	**0.33 (0.18-0.61)**	**0.20 (0.10-0.39)**	**0.15 (0.06-0.37)**	**0.33 (0.17-0.64)**	**0.20 (0.09-0.44)**	**0.35 (0.16-0.74)**	**0.41 (0.19-0.87)**	**0.41 (0.18-0.96)**

Prequit time	**0.85 (0.80-0.89)**	**0.92 (0.89-0.96)**	**0.91 (0.87-0.94)**	**0.89 (0.85-0.93)**	**0.92 (0.87-0.96)**	**0.90 (0.85-0.95)**	**0.90 (0.86-0.95)**	**0.87 (0.82-0.92)**
Number of quit attempts (ref: 1)								
2+	0.92 (0.32-2.67)	**2.94 (1.41-6.14)**	**3.38 (1.64-6.97)**	**4.40 (2.18-8.89)**	**3.12 (1.38-7.05)**	**3.13 (1.33-7.38)**	2.17 (0.82-5.75)	2.17 (0.81-5.81)
Intervention Targeting (ref: Latinos, SFTXT)								
Latinos, SFTXTesp	**0.65 (0.45-0.95)**	**0.55 (0.37-0.83)**	**0.62 (0.42-0.91)**	0.99 (0.66-1.49)	**0.62 (0.39-0.97)**	**0.53 (0.33-0.86)**	0.99 (0.61-1.62)	1.16 (0.72-1.87)

Logistic regression modeled the probability of 1= responder in response status models and 1= nonsmoker in smoking status models.
aOR: adjusted odds ratio, CI: confidence intervals
Bolded cells represent significant results.
[a]aOR was undefined.
[b]All users who selected “non-binary” or “prefer not to say” had less than 72 days between quit day and end of study date and were excluded from day 72 analyses.

Supplementary Table 7: Correlates of engagement and abstinence outcomes among SmokefreeTXT en Español (SFTXTesp) and SmokefreeTXT (SFTXT) Latino and White intervention initiators who completed the intervention (completers dataset)

	Response status							
	**Quit Day** *n*= 5758	**Day 7** *n*= 5758	**Day 14** *n*= 5758	**Day 21** *n*= 5758	**Day 28** *n*= 5758	**Day 35** *n*= 5758	**Day 42** *n*= 5758	**Day 72** *n*= 5315
	aOR (95% CI)	aOR (95% CI)	aOR (95% CI)	aOR (95% CI)	aOR (95% CI)	aOR (95% CI)	aOR (95% CI)	aOR (95% CI)
Age	**1.01 (1.01-1.02)**	**1.01 (1.01-1.02)**	**1.01 (1.01-1.02)**	**1.02 (1.02-1.03)**	**1.02 (1.01-1.03)**	**1.02 (1.02-1.03)**	**1.03 (1.02-1.03)**	**1.02 (1.02-1.03)**
Gender (ref: female)								
Male	**0.74 (0.64-0.85)**	**0.69 (0.60-0.79)**	**0.72 (0.63-0.84)**	**0.77 (0.66-0.90)**	**0.79 (0.67-0.93)**	0.88 (0.75-1.04)	**0.82 (0.68-0.98)**	**0.75 (0.63-0.88)**
Other	0.21 (0.03-1.58)	0.38 (0.09-1.66)	0.23 (0.03-1.70)	0.66 (0.15-2.86)	0.33 (0.04-2.49)	-[a]	-[a]	-[b]
Region (ref: South)								
Northeast	0.89 (0.73-1.07)	1.16 (0.97-1.39)	1.14 (0.95-1.38)	1.09 (0.90-1.33)	1.06 (0.86-1.31)	1.05 (0.84-1.30)	1.10 (0.86-1.41)	1.00 (0.80-1.25)
Midwest	**1.21 (1.01-1.44)**	1.11 (0.93-1.32)	1.15 (0.96-1.37)	**1.26 (1.04-1.51)**	**1.34 (1.11-1.63)**	**1.27 (1.04-1.55)**	**1.49 (1.19-1.86)**	1.11 (0.90-1.36)
West	1.08 (0.89-1.30)	**1.25 (1.04-1.50)**	0.93 (0.76-1.13)	1.13 (0.92-1.38)	**1.26 (1.02-1.56)**	1.20 (0.96-1.49)	**1.37 (1.07-1.74)**	1.00 (0.80-1.26)
Puerto Rico & Virgin Islands	0.99 (0.50-1.94)	0.55 (0.24-1.26)	1.00 (0.50-2.01)	0.93 (0.44-1.96)	1.09 (0.50-2.39)	0.81 (0.31-2.09)	1.30 (0.56-3.01)	1.06 (0.50-2.26)
Smoking frequency (ref: nondaily)								
Daily	0.98 (0.77-1.26)	1.05 (0.82-1.33)	1.07 (0.83-1.39)	0.87 (0.67-1.12)	0.87 (0.67-1.13)	0.85 (0.65-1.12)	1.04 (0.76-1.43)	**0.70 (0.54-0.92)**
Cigarettes smoked per day (ref: light)								
Moderate	0.85 (0.72-1.02)	**0.79 (0.67-0.93)**	0.85 (0.71-1.01)	0.88 (0.73-1.05)	**0.78 (0.65-0.94)**	**0.81 (0.66-0.98)**	**0.72 (0.58-0.89)**	**0.76 (0.62-0.93)**
Heavy	**0.48 (0.39-0.59)**	**0.45 (0.37-0.54)**	**0.45 (0.37-0.56)**	**0.48 (0.39-0.59)**	**0.48 (0.38-0.59)**	**0.49 (0.39-0.61)**	**0.50 (0.39-0.64)**	**0.52 (0.41-0.66)**
Prequit Time	**0.87 (0.86-0.88)**	**0.89 (0.88-0.90)**	**0.88 (0.87-0.90)**	**0.89 (0.88-0.90)**	**0.89 (0.87-0.90)**	**0.89 (0.88-0.91)**	**0.89 (0.87-0.91)**	**0.90 (0.88-0.92)**

Number of quit attempts (ref: 1)								
2+	**1.83 (1.27-2.64)**	**2.89 (2.10-3.97)**	**2.51 (1.78-3.54)**	**2.64 (1.87-3.73)**	**2.36 (1.64-3.41)**	**2.33 (1.60-3.39)**	**2.24 (1.47-3.41)**	**2.07 (1.39-3.08)**
Race and ethnicity (ref: Whites, SFTXT)								
Latinos, SFTXTesp	**0.51 (0.42-0.63)**	**0.41 (0.33-0.50)**	**0.47 (0.38-0.57)**	**0.51 (0.41-0.64)**	**0.46 (0.36-0.58)**	**0.37 (0.29-0.48)**	**0.60 (0.47-0.79)**	**0.64 (0.51-0.81)**
Latinos, SFTXT	**0.45 (0.36-0.56)**	**0.38 (0.30-0.47)**	**0.41 (0.32-0.52)**	**0.34 (0.26-0.44)**	**0.39 (0.30-0.51)**	**0.38 (0.29-0.50)**	**0.30 (0.21-0.43)**	**0.33 (0.25-0.45)**
**Smoking status**								
	**Quit Day** *n*= 5758	**Day 7** *n*= 5758	**Day 14** *n*= 5758	**Day 21** *n*= 5758	**Day 28** *n*= 5758	**Day 35** *n*= 5758	**Day 42** *n*= 5758	**Day 72** *n*= 5347
	aOR (95% CI)	aOR (95% CI)	aOR (95% CI)	aOR (95% CI)	aOR (95% CI)	aOR (95% CI)	aOR (95% CI)	aOR (95% CI)
Age	**1.02 (1.01-1.02)**	**1.02 (1.01-1.02)**	**1.02 (1.01-1.02)**	**1.02 (1.02-1.03)**	**1.02 (1.02-1.03)**	**1.02 (1.02-1.03)**	**1.02 (1.02-1.03)**	**1.02 (1.02-1.03)**
Gender (ref: female)								
Male	**0.75 (0.63-0.88)**	**0.71 (0.60-0.84)**	**0.74 (0.63-0.88)**	**0.82 (0.69-0.98)**	**0.77 (0.64-0.93)**	0.85 (0.71-1.03)	0.83 (0.68-1.02)	**0.77 (0.62-0.94)**
Other	0.35 (0.05-2.66)	0.69 (0.16-3.02)	0.36 (0.05-2.71)	0.94 (0.22-4.14)	0.47 (0.06-3.59)	-[a]	-[a]	-[b]
Region (ref: South)								
Northeast	1.02 (0.82-1.27)	**1.29 (1.04-1.61)**	1.09 (0.87-1.35)	1.09 (0.87-1.36)	1.06 (0.83-1.34)	1.08 (0.85-1.38)	1.17 (0.89-1.54)	0.94 (0.72-1.23)
Midwest	**1.26 (1.02-1.54)**	**1.31 (1.07-1.61)**	1.21 (0.99-1.49)	**1.31 (1.07-1.62)**	**1.34 (1.08-1.67)**	**1.36 (1.09-1.70)**	**1.49 (1.16-1.91)**	1.20 (0.94-1.53)
West	1.16 (0.93-1.45)	**1.26 (1.01-1.58)**	1.00 (0.79-1.25)	1.08 (0.85-1.36)	1.25 (0.98-1.58)	1.25 (0.98-1.60)	**1.46 (1.12-1.92)**	1.15 (0.89-1.50)
Puerto Rico & Virgin Islands	0.89 (0.34-2.32)	0.46 (0.11-1.94)	1.18 (0.48-2.87)	0.17 (0.02-1.28)	0.27 (0.04-2.02)	1.07 (0.32-3.57)	1.81 (0.68-4.80)	1.32 (0.50-3.49)
Smoking frequency (ref: nondaily)								
Daily	0.86 (0.65-1.13)	1.07 (0.80-1.43)	1.04 (0.77-1.40)	0.78 (0.59-1.04)	0.81 (0.61-1.09)	0.84 (0.62-1.14)	0.93 (0.66-1.30)	0.91 (0.65-1.28)
Cigarettes smoked per day (ref: light)								
Moderate	0.86 (0.71-1.05)	0.86 (0.70-1.05)	0.85 (0.69-1.04)	0.87 (0.71-1.07)	**0.79 (0.63-0.97)**	0.84 (0.67-1.04)	**0.76 (0.60-0.96)**	0.81 (0.64-1.03)

Heavy	**0.46 (0.36-0.57)**	**0.56 (0.44-0.70)**	**0.48 (0.38-0.60)**	**0.52 (0.41-0.65)**	**0.52 (0.41-0.66)**	**0.53 (0.41-0.68)**	**0.53 (0.41-0.70)**	**0.57 (0.43-0.75)**
Prequit Time	**0.85 (0.83-0.86)**	**0.87 (0.85-0.89)**	**0.87 (0.85-0.88)**	**0.87 (0.86-0.89)**	**0.87 (0.86-0.89)**	**0.88 (0.86-0.90)**	**0.88 (0.86-0.90)**	**0.88 (0.86-0.90)**
Number of quit attempts (ref: 1)								
2+	1.53 (0.97-2.41)	**2.49 (1.68-3.69)**	**2.66 (1.80-3.94)**	**2.89 (1.96-4.26)**	**2.79 (1.86-4.19)**	**2.97 (1.99-4.45)**	**2.26 (1.41-3.60)**	**1.92 (1.17-3.15)**
Race and ethnicity (ref: Whites, SFTXT)								
Latinos, SFTXTesp	**0.30 (0.23-0.40)**	**0.24 (0.17-0.33)**	**0.28 (0.21-0.38)**	**0.35 (0.27-0.47)**	**0.24 (0.17-0.34)**	**0.19 (0.13-0.28)**	**0.34 (0.24-0.49)**	**0.40 (0.29-0.54)**
Latinos, SFTXT	**0.44 (0.34-0.58)**	**0.37 (0.28-0.49)**	**0.40 (0.30-0.53)**	**0.33 (0.25-0.45)**	**0.34 (0.25-0.47)**	**0.34 (0.25-0.46)**	**0.33 (0.23-0.48)**	**0.32 (0.22-0.46)**

Logistic regression modeled the probability of 1= responder in response status models and 1= nonsmoker in smoking status models.
aOR: adjusted odds ratio, CI: confidence intervals
Bolded cells represent significant results.
[a]aOR was undefined.
[b]All users who selected "non-binary" or "prefer not to say" had less than 72 days between quit day and end of study date and were excluded from day 72 analyses.

Supplementary Table 8: Response rates and point-prevalence abstinence among SmokefreeTXT en Español (SFTXTesp) and SmokefreeTXT (SFTXT) intervention initiators (complete case dataset, *N*= 9,367)

	Did not respond, *n*	Responded		Response rate denominator, *n*	Response rate, %	Abstinence rate denominator, *n*	Abstinence, %
		Abstinent, *n*	Not abstinent, *n*				
**Quit day**							
Latinos, SFTXTesp	728	51	76	855	14.85	855	5.96
Latinos, SFTXT	1392	112	73	1577	11.73	1577	7.10
Whites, SFTXT	5205	1205	525	6935	24.95	6935	17.38
Overall	7325	1368	674	9367	21.80	9367	14.60
**Day 7**							
Latinos, SFTXTesp	623	50	61	734	15.12	855	5.85
Latinos, SFTXT	999	86	68	1153	13.36	1577	5.45
Whites, SFTXT	3594	948	570	5112	29.69	6935	13.67
Overall	5216	1084	699	6999	25.48	9367	11.57
**Day 14**							
Latinos, SFTXTesp	600	35	45	680	11.76	855	4.09
Latinos, SFTXT	953	78	37	1068	10.77	1577	4.95
Whites, SFTXT	3387	826	328	4541	25.41	6935	11.91
Overall	4940	939	410	6289	21.45	9367	10.02
**Day 21**							
Latinos, SFTXTesp	586	42	38	666	12.01	855	4.91
Latinos, SFTXT	942	63	26	1031	8.63	1577	3.99
Whites, SFTXT	3334	746	225	4305	22.56	6935	10.76
Overall	4862	851	289	6002	18.99	9367	9.09
**Day 28**							
Latinos, SFTXTesp	593	23	31	647	8.35	855	2.69
Latinos, SFTXT	925	56	25	1006	8.05	1577	3.55
Whites, SFTXT	3301	638	164	4103	19.55	6935	9.20
Overall	4819	717	220	5756	16.28	9367	7.65
**Day 35**							
Latinos, SFTXTesp	597	19	21	637	6.28	855	2.22
Latinos, SFTXT	924	48	20	992	6.85	1577	3.04
Whites, SFTXT	3228	615	129	3972	18.73	6935	8.87
Overall	4749	682	170	5601	15.21	9367	7.28

**Day 42**							
Latinos, SFTXTesp	569	27	23	619	8.08	855	3.16
Latinos, SFTXT	932	33	3	968	3.72	1577	2.09
Whites, SFTXT	3321	430	82	3833	13.36	6935	6.20
Overall	4822	490	108	5420	11.03	9367	5.23
**Day 72**							
Latinos, SFTXTesp	537	31	36	604	11.09	834	3.72
Latinos, SFTXT	807	32	19	858	5.94	1416	2.26
Whites, SFTXT	2879	444	177	3500	17.74	6423	6.91
Overall	4223	507	232	4962	14.89	8673	5.85

Supplementary Table 9: Correlates of engagement and abstinence outcomes among SmokefreeTXT en Español (SFTXTesp) and SmokefreeTXT (SFTXT) Latino intervention initiators (complete case dataset)

	Response status							
	**Quit Day** *n*= 2432	**Day 7** *n*= 1887	**Day 14** *n*= 1748	**Day 21** *n*= 1697	**Day 28** *n*= 1653	**Day 35** *n*= 1629	**Day 42** *n*= 1587	**Day 72** *n*= 1462
	aOR (95% CI)	aOR (95% CI)	aOR (95% CI)	aOR (95% CI)	aOR (95% CI)	aOR (95% CI)	aOR (95% CI)	aOR (95% CI)
Age	1.01 (1.00-1.02)	1.01 (1.00-1.02)	1.00 (0.99-1.02)	1.01 (0.99-1.02)	1.01 (0.99-1.02)	1.00 (0.99-1.02)	1.01 (0.99-1.03)	1.00 (0.98-1.02)
Gender (ref: female)								
Male	0.80 (0.62-1.03)	0.87 (0.66-1.14)	**0.59 (0.42-0.82)**	0.90 (0.64-1.25)	0.94 (0.65-1.36)	0.91 (0.61-1.37)	0.83 (0.53-1.31)	0.68 (0.45-1.03)
Other	0.34 (0.05-2.61)	0.49 (0.06-3.78)	-[a]	0.84 (0.11-6.71)	-[a]	-[a]	-[a]	-[b]
Region (ref: South)								
Northeast	1.03 (0.74-1.45)	1.18 (0.82-1.70)	1.09 (0.72-1.65)	0.96 (0.61-1.51)	1.20 (0.72-2.00)	1.57 (0.91-2.69)	0.57 (0.28-1.17)	1.07 (0.64-1.78)
Midwest	0.78 (0.51-1.20)	0.94 (0.60-1.48)	1.27 (0.79-2.03)	0.96 (0.56-1.64)	1.44 (0.81-2.57)	1.25 (0.64-2.43)	1.36 (0.70-2.61)	0.48 (0.22-1.05)
West	1.13 (0.83-1.55)	1.15 (0.81-1.63)	0.68 (0.44-1.06)	1.03 (0.67-1.58)	1.44 (0.90-2.30)	1.49 (0.88-2.51)	1.10 (0.62-1.94)	0.86 (0.51-1.44)
Puerto Rico & Virgin Islands	0.96 (0.50-1.83)	0.71 (0.32-1.56)	1.23 (0.58-2.60)	1.14 (0.52-2.50)	1.03 (0.38-2.79)	1.21 (0.40-3.66)	1.41 (0.54-3.65)	1.38 (0.59-3.22)
Smoking frequency (ref: nondaily)								
Daily	**1.59 (1.05-2.41)**	1.20 (0.79-1.84)	1.10 (0.67-1.80)	0.92 (0.56-1.52)	1.06 (0.60-1.85)	1.39 (0.71-2.72)	1.26 (0.60-2.64)	**0.58 (0.34-0.99)**
Cigarettes smoked per day (ref: light)								
Moderate	**0.73 (0.55-0.96)**	**0.53 (0.39-0.71)**	0.73 (0.52-1.03)	0.85 (0.59-1.21)	**0.60 (0.40-0.89)**	0.65 (0.42-1.01)	**0.53 (0.32-0.87)**	0.67 (0.44-1.04)
Heavy	**0.18 (0.12-0.26)**	**0.17 (0.11-0.27)**	**0.15 (0.09-0.26)**	**0.19 (0.11-0.33)**	**0.19 (0.10-0.34)**	**0.23 (0.13-0.43)**	**0.21 (0.10-0.44)**	**0.21 (0.11-0.41)**
Prequit time	**0.90 (0.88-0.93)**	**0.93 (0.91-0.96)**	**0.92 (0.89-0.95)**	**0.92 (0.89-0.96)**	**0.92 (0.89-0.96)**	**0.93 (0.89-0.97)**	**0.94 (0.89-0.98)**	**0.91 (0.87-0.95)**

Number of quit attempts (ref: 1)								
2+	0.97 (0.47-2.03)	**2.48 (1.31-4.70)**	**2.33 (1.09-4.97)**	**3.26 (1.56-6.80)**	**3.40 (1.58-7.33)**	**2.49 (1.05-5.89)**	**3.86 (1.59-9.38)**	**3.68 (1.55-8.75)**
Intervention targeting (ref: Latinos, SFTXT)								
Latinos, SFTXTesp	0.94 (0.72-1.22)	0.90 (0.68-1.20)	0.77 (0.56-1.07)	1.05 (0.75-1.48)	0.78 (0.53-1.14)	0.69 (0.45-1.05)	**1.73 (1.08-2.76)**	**1.58 (1.05-2.38)**

**Smoking status**								
	**Quit Day** *n*= 2432	**Day 7** *n*= 2432	**Day 14** *n*= 2432	**Day 21** *n*= 2432	**Day 28** *n*= 2432	**Day 35** *n*= 2432	**Day 42** *n*= 2432	**Day 72** *n*= 2250
	aOR (95% CI)	aOR (95% CI)	aOR (95% CI)	aOR (95% CI)	aOR (95% CI)	aOR (95% CI)	aOR (95% CI)	aOR (95% CI)
Age	1.01 (1.00-1.03)	1.01 (1.00-1.02)	1.01 (0.99-1.02)	1.01 (1.00-1.03)	1.00 (0.99-1.02)	1.00 (0.99-1.02)	1.01 (0.99-1.03)	1.00 (0.98-1.02)
Gender (ref: female)								
Male	**0.66 (0.47-0.94)**	1.07 (0.74-1.53)	0.73 (0.48-1.11)	0.90 (0.60-1.37)	1.10 (0.69-1.76)	0.95 (0.57-1.58)	0.72 (0.42-1.25)	**0.55 (0.31-0.96)**
Other	0.49 (0.06-3.78)	0.80 (0.10-6.19)	-[a]	0.97 (0.12-7.57)	-[a]	-[a]	-[a]	-[b]
Region (ref: South)								
Northeast	0.94 (0.59-1.48)	**1.60 (1.02-2.52)**	1.10 (0.65-1.87)	1.05 (0.62-1.78)	1.64 (0.90-3.00)	1.53 (0.77-3.05)	0.61 (0.27-1.38)	0.84 (0.41-1.70)
Midwest	1.00 (0.59-1.72)	1.40 (0.81-2.42)	**1.79 (1.02-3.12)**	1.25 (0.69-2.25)	1.51 (0.73-3.09)	1.77 (0.82-3.81)	0.89 (0.37-2.10)	0.48 (0.16-1.40)
West	1.20 (0.79-1.81)	0.88 (0.54-1.45)	0.82 (0.47-1.40)	0.79 (0.47-1.35)	1.30 (0.71-2.37)	1.54 (0.80-2.96)	1.08 (0.57-2.07)	1.10 (0.58-2.05)
Puerto Rico & Virgin Islands	1.42 (0.63-3.19)	0.84 (0.29-2.44)	1.32 (0.49-3.55)	0.19 (0.03-1.45)	-[a]	1.21 (0.27-5.46)	1.91 (0.68-5.34)	1.16 (0.38-3.53)
Smoking frequency (ref: nondaily)								
Daily	1.23 (0.73-2.07)	1.38 (0.78-2.44)	1.38 (0.75-2.56)	0.84 (0.47-1.49)	1.37 (0.66-2.84)	1.21 (0.56-2.63)	1.24 (0.54-2.82)	0.68 (0.34-1.36)
Cigarettes smoked per day (ref: light)								
Moderate	0.70 (0.49-1.02)	**0.53 (0.35-0.79)**	**0.58 (0.38-0.89)**	0.67 (0.43-1.05)	**0.55 (0.33-0.92)**	0.59 (0.34-1.04)	**0.54 (0.29-0.98)**	1.06 (0.61-1.86)
Heavy	**0.23 (0.14-0.37)**	**0.18 (0.10-0.31)**	**0.08 (0.04-0.18)**	**0.22 (0.12-0.41)**	**0.16 (0.08-0.33)**	**0.24 (0.12-0.49)**	**0.29 (0.14-0.63)**	**0.29 (0.12-0.70)**

Prequit time	**0.89 (0.85-0.92)**	**0.94 (0.90-0.97)**	**0.94 (0.90-0.98)**	**0.92 (0.88-0.96)**	**0.95 (0.90-0.99)**	**0.94 (0.89-0.99)**	**0.93 (0.88-0.99)**	**0.90 (0.84-0.96)**
Number of quit attempts (ref: 1)								
2+	0.38 (0.09-1.60)	1.85 (0.81-4.20)	**2.33 (1.01-5.37)**	**3.14 (1.43-6.91)**	**2.72 (1.11-6.66)**	**3.23 (1.31-7.95)**	2.42 (0.83-7.03)	2.53 (0.86-7.46)
Intervention targeting (ref: Latinos, SFTXT)								
Latinos, SFTXTesp	**0.60 (0.42-0.87)**	0.80 (0.55-1.17)	**0.54 (0.35-0.83)**	1.00 (0.66-1.52)	**0.58 (0.35-0.96)**	**0.56 (0.32-0.98)**	1.15 (0.66-1.99)	1.32 (0.78-2.24)

Logistic regression modeled the probability of 1= responder in response status models and 1= nonsmoker in smoking status models.
aOR: adjusted odds ratio, CI: confidence intervals
Bolded cells represent significant results.
[a]aOR was undefined.
[b]All users who selected "non-binary" or "prefer not to say" had less than 72 days between quit day and end of study date and were excluded from day 72 analyses.

Supplementary Table 10. Correlates of engagement and abstinence outcomes among SmokefreeTXT en Español (SFTXTesp) and SmokefreeTXT (SFTXT) Latino and White intervention initiators (complete case dataset)

	**Response status**							
	**Quit Day** *n*= 9367	**Day 7** *n*= 6999	**Day 14** *n*= 6289	**Day 21** *n*= 6002	**Day 28** *n*= 5756	**Day 35** *n*= 5601	**Day 42** *n*= 5420	**Day 72** *n*= 4962
	aOR (95% CI)	aOR (95% CI)	aOR (95% CI)	aOR (95% CI)	aOR (95% CI)	aOR (95% CI)	aOR (95% CI)	aOR (95% CI)
Age	**1.02 (1.02-1.02)**	**1.02 (1.01-1.02)**	**1.01 (1.01-1.02)**	**1.02 (1.02-1.03)**	**1.02 (1.02-1.03)**	**1.02 (1.02-1.03)**	**1.03 (1.02-1.03)**	**1.02 (1.02-1.03)**
Gender (ref: female)								
Male	**0.73 (0.65-0.82)**	**0.73 (0.64-0.82)**	**0.75 (0.65-0.86)**	**0.75 (0.65-0.87)**	**0.79 (0.68-0.93)**	0.87 (0.74-1.03)	**0.79 (0.65-0.96)**	**0.72 (0.60-0.86)**
Other	**0.22 (0.05-0.91)**	0.27 (0.06-1.15)	0.39 (0.09-1.68)	0.51 (0.12-2.24)	0.26 (0.03-1.99)	-[a]	-[a]	-[b]
Region (ref: South)								
Northeast	0.87 (0.75-1.01)	1.12 (0.96-1.31)	1.14 (0.96-1.36)	1.03 (0.85-1.25)	1.11 (0.90-1.37)	1.10 (0.88-1.36)	1.10 (0.85-1.42)	1.05 (0.83-1.32)
Midwest	1.12 (0.98-1.28)	**1.17 (1.01-1.36)**	1.16 (0.98-1.37)	**1.25 (1.05-1.50)**	**1.35 (1.12-1.64)**	**1.32 (1.08-1.61)**	**1.50 (1.19-1.89)**	1.18 (0.96-1.47)
West	1.13 (0.98-1.30)	**1.23 (1.05-1.45)**	0.96 (0.79-1.15)	1.14 (0.94-1.39)	**1.27 (1.03-1.57)**	**1.26 (1.01-1.56)**	**1.40 (1.09-1.81)**	1.08 (0.85-1.36)
Puerto Rico & Virgin Islands	0.93 (0.49-1.74)	0.67 (0.31-1.44)	1.24 (0.60-2.54)	1.16 (0.55-2.47)	0.90 (0.35-2.36)	0.99 (0.34-2.87)	1.57 (0.63-3.91)	1.49 (0.66-3.35)
Smoking Frequency (ref: nondaily)								
Daily	1.05 (0.86-1.27)	1.04 (0.84-1.28)	1.18 (0.93-1.51)	0.97 (0.76-1.25)	0.96 (0.74-1.26)	0.92 (0.70-1.21)	1.03 (0.75-1.42)	**0.72 (0.55-0.95)**
Cigarettes per day (ref: light)								
Moderate	0.90 (0.79-1.02)	**0.77 (0.67-0.89)**	0.87 (0.74-1.02)	0.85 (0.72-1.00)	**0.74 (0.62-0.89)**	**0.80 (0.66-0.97)**	**0.69 (0.56-0.86)**	**0.73 (0.60-0.90)**
Heavy	**0.47 (0.41-0.55)**	**0.48 (0.41-0.56)**	**0.48 (0.40-0.58)**	**0.48 (0.39-0.58)**	**0.48 (0.39-0.59)**	**0.48 (0.39-0.60)**	**0.48 (0.37-0.61)**	**0.50 (0.40-0.63)**
Prequit Time	**0.89 (0.88-0.90)**	**0.90 (0.89-0.91)**	**0.89 (0.88-0.90)**	**0.90 (0.88-0.91)**	**0.89 (0.87-0.90)**	**0.89 (0.88-0.91)**	**0.89 (0.87-0.91)**	**0.90 (0.88-0.92)**

Number of quit attempts (ref: 1)								
2+	**1.50 (1.13-2.00)**	**2.20 (1.63-2.96)**	**2.16 (1.55-3.02)**	**2.28 (1.60-3.23)**	**2.23 (1.54-3.25)**	**2.32 (1.58-3.40)**	**2.21 (1.42-3.45)**	**1.91 (1.24-2.94)**
Race and ethnicity (ref: Whites, SFTXT)								
Latinos, SFTXTesp	**0.50 (0.41-0.62)**	**0.41 (0.33-0.52)**	**0.37 (0.28-0.48)**	**0.45 (0.35-0.59)**	**0.37 (0.27-0.50)**	**0.28 (0.20-0.39)**	**0.56 (0.40-0.77)**	**0.56 (0.42-0.74)**
Latinos, SFTXT	**0.46 (0.39-0.55)**	**0.41 (0.34-0.50)**	**0.41 (0.33-0.51)**	**0.38 (0.30-0.48)**	**0.42 (0.33-0.54)**	**0.37 (0.28-0.49)**	**0.29 (0.20-0.41)**	**0.33 (0.24-0.44)**
**Smoking status**								
	**Quit Day** *n*= 9367	**Day 7** *n*= 9367	**Day 14** *n*= 9367	**Day 21** *n*= 9367	**Day 28** *n*= 9367	**Day 35** *n*= 9367	**Day 42** *n*= 9367	**Day 72** *n*= 8673
	aOR (95% CI)	aOR (95% CI)	aOR (95% CI)	aOR (95% CI)	aOR (95% CI)	aOR (95% CI)	aOR (95% CI)	aOR (95% CI)
Age	**1.02 (1.02-1.03)**	**1.03 (1.02-1.03)**	**1.02 (1.02-1.03)**	**1.03 (1.02-1.03)**	**1.03 (1.02-1.03)**	**1.03 (1.02-1.03)**	**1.03 (1.02-1.04)**	**1.03 (1.02-1.03)**
Gender (ref: female)								
Male	**0.76 (0.67-0.87)**	**0.73 (0.63-0.84)**	**0.77 (0.67-0.90)**	**0.81 (0.69-0.95)**	**0.81 (0.68-0.96)**	0.86 (0.73-1.03)	0.84 (0.69-1.03)	**0.76 (0.62-0.93)**
Other	0.38 (0.09-1.59)	0.45 (0.11-1.91)	0.27 (0.04-1.99)	0.68 (0.16-2.88)	0.36 (0.05-2.65)	-[a]	-[a]	-[b]
Region (ref: South)								
Northeast	0.91 (0.77-1.08)	**1.37 (1.14-1.64)**	**1.24 (1.03-1.51)**	1.13 (0.92-1.38)	1.16 (0.93-1.45)	1.25 (0.99-1.56)	1.27 (0.97-1.66)	1.07 (0.82-1.39)
Midwest	1.16 (0.99-1.35)	**1.31 (1.11-1.56)**	**1.29 (1.08-1.55)**	**1.36 (1.13-1.64)**	**1.38 (1.13-1.69)**	**1.44 (1.17-1.77)**	**1.54 (1.21-1.96)**	**1.31 (1.03-1.66)**
West	1.10 (0.93-1.31)	1.20 (0.99-1.45)	1.07 (0.87-1.31)	1.08 (0.87-1.33)	1.24 (0.99-1.55)	**1.33 (1.06-1.67)**	**1.52 (1.17-1.98)**	1.26 (0.98-1.64)
Puerto Rico & Virgin Islands	1.38 (0.63-3.00)	0.77 (0.27-2.19)	1.32 (0.51-3.43)	0.21 (0.03-1.53)	-[a]	0.97 (0.23-4.15)	2.42 (0.91-6.47)	1.48 (0.51-4.30)
Smoking frequency (ref: nondaily)								
Daily	0.89 (0.71-1.10)	1.12 (0.87-1.43)	1.13 (0.87-1.47)	0.94 (0.72-1.22)	0.95 (0.72-1.26)	0.93 (0.70-1.24)	0.94 (0.68-1.31)	0.97 (0.70-1.35)
Cigarettes smoked per day (ref: light)								
Moderate	0.87 (0.75-1.02)	**0.81 (0.69-0.96)**	0.84 (0.71-1.00)	**0.81 (0.68-0.97)**	**0.75 (0.62-0.91)**	**0.81 (0.66-0.98)**	**0.72 (0.58-0.91)**	**0.78 (0.62-0.98)**

Heavy	**0.46 (0.39-0.55)**	**0.52 (0.43-0.63)**	**0.48 (0.39-0.59)**	**0.48 (0.39-0.59)**	**0.51 (0.41-0.64)**	**0.51 (0.40-0.64)**	**0.50 (0.39-0.65)**	**0.55 (0.42-0.71)**
Prequit Time	**0.87 (0.85-0.88)**	**0.89 (0.88-0.91)**	**0.90 (0.89-0.92)**	**0.91 (0.89-0.93)**	**0.90 (0.88-0.92)**	**0.91 (0.89-0.93)**	**0.91 (0.89-0.93)**	**0.91 (0.89-0.93)**
Number of quit attempts (ref: 1)								
2+	1.34 (0.95-1.88)	**1.65 (1.18-2.32)**	**1.69 (1.19-2.40)**	**1.71 (1.19-2.45)**	**2.02 (1.40-2.93)**	**2.22 (1.55-3.20)**	**1.81 (1.16-2.82)**	1.45 (0.90-2.34)
Race and ethnicity (ref: Whites, SFTXT)								
Latinos, SFTXTesp	**0.28 (0.20-0.38)**	**0.40 (0.30-0.55)**	**0.31 (0.21-0.44)**	**0.46 (0.33-0.64)**	**0.30 (0.19-0.46)**	**0.23 (0.14-0.38)**	**0.45 (0.30-0.70)**	**0.52 (0.35-0.77)**
Latinos, SFTXT	**0.41 (0.34-0.51)**	**0.42 (0.33-0.53)**	**0.45 (0.35-0.57)**	**0.40 (0.31-0.53)**	**0.42 (0.31-0.55)**	**0.37 (0.27-0.50)**	**0.36 (0.25-0.52)**	**0.34 (0.24-0.50)**

Logistic regression modeled the probability of 1= responder in response status models and 1= nonsmoker in smoking status models.
aOR: adjusted odds ratio, CI: confidence intervals
Bolded cells represent significant results.
[a]aOR was undefined.
[b]All users who selected "non-binary" or "prefer not to say" had less than 72 days between quit day and end of study date and were excluded from day 72 analyses.

Supplementary Table 11: Correlates of enrolling in SmokefreeTXT en Español (i.e., being an SFTXTesp user, *N*= 3863)

	aOR	LCL	UCL
Age	**1.01**	**1.01**	**1.02**
Gender (ref: female)			
Male	**1.52**	**1.31**	**1.76**
Other	-[a]	-[a]	-[a]
Region (ref: South)			
Northeast	**0.71**	**0.58**	**0.87**
Midwest	**0.73**	**0.58**	**0.92**
West	**0.69**	**0.57**	**0.84**
Puerto Rico & Virgin Islands	**5.60**	**3.26**	**9.62**
Smoking Frequency (ref: nondaily)			
Daily	1.24	0.98	1.57
Cigarettes smoked per day (ref: light)			
Moderate	1.06	0.87	1.29
Heavy	**0.16**	**0.13**	**0.20**
Prequit time	1.00	0.99	1.02
Number of quit attempts (ref: 1)			
2+	1.23	0.86	1.77

LCL: Lower confidence level; UCL: Upper confidence level.
Bolded cells represent significant results.
[a]"Non-binary" or "prefer not to say" options were not available to SFTXTesp users, resulting in an undefined aOR. For sensitivity analysis, we ran two alternate models. Those who selected "non-binary/prefer not to say" were classified as females in the first model and as "males" in the second model. All significant and non-significant associations reported above remained consistent in our two alternate models.
